# Strain sharing and persistence of microbial pathogens colonizing the skin of residents in a regional nursing home network

**DOI:** 10.1038/s41467-026-74611-x

**Published:** 2026-06-18

**Authors:** Yaovi M. G. Hounmanou, Gabrielle M. Gussin, Sean Conlan, Raveena D. Singh, Clay Deming, Diana M. Proctor, Marco Teixeira, Ashlee M. Earl, Colin J. Worby, Heidi H. Kong, Susan S. Huang, Julia A. Segre

**Affiliations:** 1 Microbial Genomics Section, NHGRI, NIH, Bethesda, MD USA; 2 Department of Veterinary and Animal Sciences, University of Copenhagen, Frederiksberg, Denmark; 3 Division of Infectious Diseases, University of California Irvine School of Medicine, Irvine, CA USA; 4 Department of Microbiology and Molecular Genetics, McGovern Medical School, The University of Texas Health Science Center at Houston, Houston, TX USA; 5 Infectious Disease & Microbiome Program, Broad Institute, Cambridge, MA USA; 6 Delft Bioinformatics Lab, Department of Intelligent Systems, Delft University of Technology, Delft, The Netherlands; 7 Cutaneous Microbiome and Inflammation Section, NIAMS, NIH, Bethesda, MD USA; 8 Present Address: Research Unit for Applied Microbiology and Pharmacology of Natural Substances, Polytechnic School of Abomey-Calavi, University of Abomey-Calavi, Abomey-Calavi, Benin

**Keywords:** Microbial communities, Microbial genetics

## Abstract

Antimicrobial resistance (AMR) is a health threat disproportionately affecting nursing home (NH) residents. Surveillance and infection control in NHs are restricted to nares or perirectal cultures, overlooking skin colonization and multidrug-resistant organisms (MDROs) not recovered by selective media. Here, within the PROTECT trial NCT03118232, we show, that NH residents’ skin serves as a reservoir of transmissible MDROs. We analyzed 207 groin and axilla swabs from 38 residents across 15 California NHs using metagenomics, culturing, and genome sequencing. Culture detected MDROs in 10 of 38 residents (26.3%), including 4 (10.5%) with ESBL-producing *Escherichia coli* sequence type (ST)131/ST648 and 7 (18.4%) with methicillin-resistant *Staphylococcus aureus*. Skin microbiome analysis by metagenome-assembled genomes identified broader MDRO colonization, including 27 (71.1%) with *E. coli* ST93, 14 (36.8%) with *Staphylococcus epidermidis* ST2, 16 (42.1%) with *Proteus mirabilis*, 7 (18.4%) with *Providencia stuartii*, 7 (18.4%) with *Enterococcus faecalis*, and 5 (13.2%) with *Pseudomonas aeruginosa*. Colonization persisted after bathing. Clonal *E. coli* ST93 was shared by 27 residents across 9 facilities, and 5 resident pairs carried clonally related strains of ≥2 MDRO species, suggesting polymicrobial transmission. We confirmed skin as a reservoir of MDROs, utilizing metagenomics to detect colonization and transmission pathways, supporting AMR surveillance in long-term care.

## Abstract (web summary)

Here, by combining skin metagenomics, culturing and isolate genome sequencing, the authors show that the skin of nursing home residents harbors persistent and transmissible drug-resistant bacteria with implications for improving clinical surveillance

## Introduction

Antimicrobial resistance (AMR) threatens decades of medical progress, rendering once-treatable infections increasingly difficult or impossible to manage. Multidrug-resistant organisms (MDROs) colonizing and infecting patients undermine treatment efficacy, increase morbidity and mortality, and escalate healthcare costs. In 2021, an estimated 4.71 million deaths were associated with bacterial AMR, with global disability-adjusted life years projected to reach 46.5 million by 2050^1^. The economic toll of direct healthcare costs associated with AMR is estimated at 66 billion USD and predicted to reach 159 billion USD without improved interventions^2^. Older adults are disproportionately affected, with AMR-related deaths increasing by more than 80% in those aged 70 years and older between 1990 and 2021^1^. Many of these vulnerable individuals reside in nursing homes (NH)^3^.

Nursing homes are increasingly recognized as persistent reservoirs for MDROs^4^. In the USA, more than 1.3 million people live in 15,300 nursing homes, where MDRO prevalence is four to six times higher than in hospitals^3,5^. Prolonged stays, frequent antibiotic use, hospital transfers, and close-contact communal living amplify opportunities for colonization and onward transmission^6^. Colonization is often asymptomatic but increases the risk of infection, making detection essential for prevention. Most studies of MDRO carriage in NHs have relied primarily on culture-based detection from perirectal sites, providing only a partial view of MDRO ecology and transmission risk^7,8^. These approaches overlook skin colonization, fail to capture non-resistant or co-colonizing strains on selective media, and lack strain-level resolution of bacterial diversity and persistence.

Recent shotgun metagenomic data primarily of residents in a single nursing home showed that the skin can harbor MDROs, including *Candida auris* and ESKAPE pathogens (*Enterococcus faecium*, *Staphylococcus aureus*, *K*. *pneumoniae*, *A*. *baumannii*, *P*. *aeruginosa*, *Enterobacter* species)^9^. The inguinal crease and axilla are recognized hotspots for microbial colonization and potential transmission^7,10,11^. As moist, occluded niches with frequent contact with textiles and caregivers, they represent epidemiologically relevant sites of microbial colonization amongst NH residents. Yet, AMR surveillance focuses almost exclusively on nares and perirectal sites and relies on culture-based methods that can miss co-colonizing strains, morphologically similar lineages, or organisms lacking expected resistance phenotypes^10^. Shotgun metagenomics analysis enables recovery of metagenome-assembled genome (MAGs)^12^, providing strain-level resolution directly from complex samples^10,13^. Integrated genomic approaches combining culturomics and metagenomics now offer an unprecedented ability to track MDRO persistence and spread. Few studies have performed skin microbiome analyses of NH residents^9,14^ and none have combined metagenomics with bacterial isolate genome sequencing to study MDRO skin ecology. This study therefore, advances the field by applying an integrated genomic approach to reveal the extent, persistence, and strain-sharing patterns of MDRO skin colonization in nursing home residents. Project PROTECT trial, a cluster-randomized study of nursing homes in California, which demonstrated that MDRO colonization was highly prevalent in NH residents and strongly associated with increased hospital transfers^5^. During this trial, skin swabs were collected from a convenience sample of residents for bacterial isolation and microbiome analysis.

Here, we show that integration of shotgun metagenomics and isolate genome sequencing identifies extensive MDRO colonization, persistence, and transmission among 38 residents in 15 nursing homes. We define the extent of MDRO skin colonization, characterize inter-resident and inter-facility strain sharing, resolve the resistome structure, and assess strain persistence. Our findings reveal cryptic, skin colonization, and polymicrobial strain sharing of MDROs among nursing home residents, informing potential future infection control strategies for long-term care settings.

## Results

### Selective culture reveals diverse MDROs on NH residents’ skin

To test for skin carriage of MDROs on nursing home residents, the axilla and groin of 38 residents of 15 NHs (2–3 residents per NH) were sampled and cultured on antibiotic selective media. Of the 10 NH residents (26.3%) with skin MDRO carriage, five residents (13.2%) carried extended-spectrum β-lactamase (ESBL)-producing gram-negative bacteria. Three residents (7.9%) in 3 different NHs carried *E. coli* sequence type (ST)131, a globally disseminated uropathogenic ESBL lineage, one resident co-carried ESBL-producing *E. coli* ST648 and *Providencia stuartii* and the fifth resident carried ESBL-producing *Proteus mirabilis*. Vancomycin-resistant *Enterococcus faecalis* (VRE) was recovered from 2 residents (5.3%), including ST109 and ST778 lineages (Table 1). In addition, methicillin-resistant *Staphylococcus aureus* (MRSA) was cultured from the skin of seven residents (18.4%). These findings confirm that skin can act as a reservoir for clinically relevant, antimicrobial-resistant organisms, with potential implications for infection risk and resident-to-resident dissemination.

**Table 1:** Skin-colonizing MDROs isolated from NH residents in this study

Species	ST	#NHs	Residents colonized	Phenotypic profile
*Escherichia coli*	ST131	3 (20%)	3 (7.9%)	ESBL
	ST648	1 (6.7%)	1 (2.6%)	ESBL
*Enterococcus faecalis*	ST109	1 (6.7%)	1 (2.6%)	VRE
	ST778	1 (6.7%)	1 (2.6%)	VRE
*Proteus mirabilis*	NA	1 (6.7%)	1 (2.6%)	ESBL
*Providencia stuartii*	ST83	1 (6.7%)	1 (2.6%)	ESBL
*Staphylococcus aureus*	-	6 (40%)	7 (18.4%)	MRSA^a^
*Overall Skin MDRO carriage*		7 (46.7%)	10 (26.3%)	

^a^The MRSA isolates were not sequenced as part of this observational study.

### Opportunistic pathogens persist within a dysbiotic skin microbiome

**Fig. 1:**
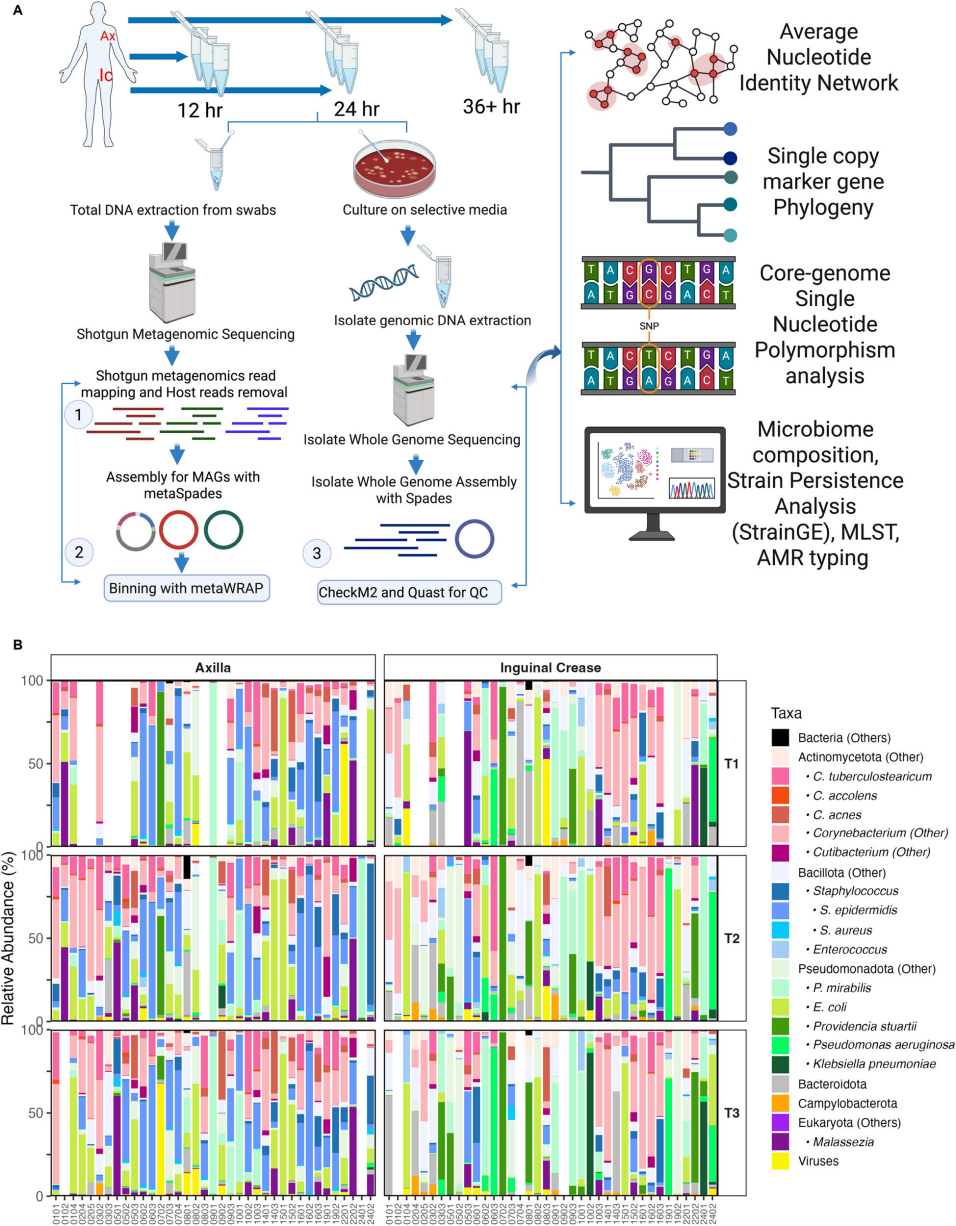
Overview of study design and skin microbiome composition analysis. **A** Study workflow for sample collection, sequencing, and genomic analysis of skin swabs collected from nursing home residents. **B** Stacked bar plots show the relative microbial abundances of individual nursing home residents, at two skin sites (axilla and inguinal crease) at three visits (Timepoint (T), T1, T2, T3). **A** was created using Biorender under publication license Segre, J. (2026) https://BioRender.com/krcae4k.

To place these culture-based findings in broader ecological context, we next analyzed the skin microbiome of these 38 residents from 15 NHs from two epidemiologically relevant skin sites (inguinal crease (Ic) and axilla (Ax)), sampled at 2–3 visits per resident between 12 h and 48 h after bathing (Fig. 1A). In total we sequenced and analyzed 223 samples including 207 skin swabs, and 16 sample controls (Supplementary Data 1 and Supplementary Fig. 1A). Analysis of the skin shotgun metagenomic data revealed a predominantly bacterial skin microbiome with typical commensal taxa (*Cutibacterium, Corynebacterium, Staphylococcus* spp.), coexisting with opportunistic pathogens such as *Enterococci, E. coli, P. mirabilis, P. aeruginosa* and *Providencia* spp that are not expected in a normal skin microbiome. Fungal (*Malassezia* spp.) and viral taxa (Betapapillomavirus, Uroviricota phages) were consistently detected but at low relative abundances (Fig. 1B).

Across samples, the overall composition of NH residents’ skin microbiome appeared broadly similar between body sites and over time, with no significant differences detected in Shannon diversity between Ic and Ax samples (*p* > 0.05). Taken together, these findings point to the presence of persistent opportunistic pathogen populations on nursing-home residents’ skin rather than large compositional shifts over the short sampling period. We therefore reconstructed MAGs to resolve organism-specific colonization patterns at higher genomic resolution.

### MAG recovery reveals extensive skin colonization by opportunistic pathogens

**Fig. 2:**
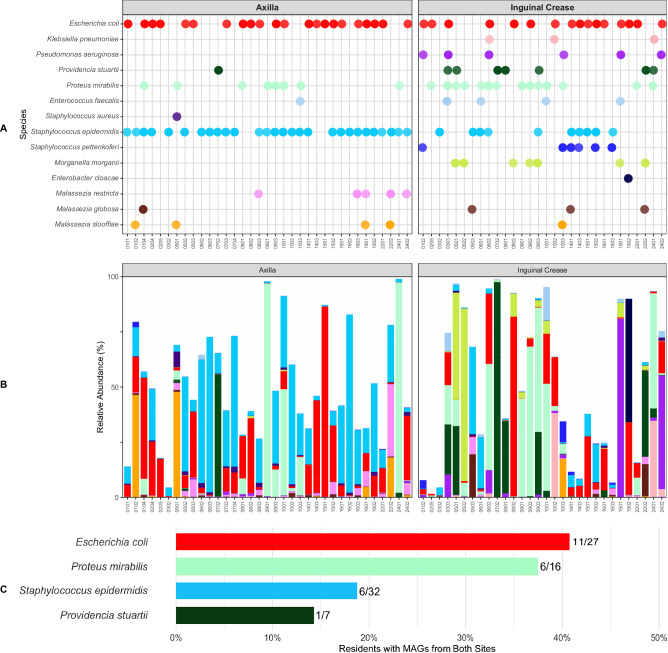
MDRO colonization of nursing home residents’ skin characterized by MAG recovery and abundance. **A** Recovery per resident at any timepoint of near-complete bacterial and fungal MAGs colored for representative species. Detailed overview of MAGs per resident per species are displayed in Supplementary Fig. 2A. **B** Relative abundance of same representative bacteria and fungi in shotgun metagenomic sequencing samples. Colors are defined in (**A**). **C** Frequency of MAG recovery from both inguinal crease and axilla of the same resident.

From the skin shotgun metagenomic data generated from 207 samples, we recovered 1185 (1160 bacterial and 25 fungal) MAGs, with a median of four MAGs per sample. Taxonomic classification revealed both skin commensals and clinically relevant microbes such as *Escherichia coli* (*n* = 68 MAGs; 60 near-complete), *Staphylococcus epidermidis* (*n* = 85; 77 near-complete), *Proteus mirabilis* (*n* = 40; 34 near-complete), *Providencia stuartii* (*n* = 17; 16 near-complete), and *Pseudomonas aeruginosa* (*n* = 11; 10 near-complete) (Fig. 2A; Supplementary Figs. 1B and 2A). Fungal MAGs were largely *Malassezia* spp., consistent with typical skin mycobiota (Fig. 2A; Supplementary Fig. S2B). MAG abundances correlated with read-level detection across samples (Fig. 2B), confirming analytic robustness.

Dual-site colonization of a resident was common for *E. coli*, with concurrent detection in axilla and inguinal crease in 11 of 27 colonized residents (40.7%), and for *P. mirabilis* in 6 of 16 residents (37.5%). *S. epidermidis* and *P. stuartii* displayed lower dual-site co-occurrence rates (18.8% and 14.3%, respectively) (Fig. 2C). These findings suggest that high-burden colonizers can simultaneously occupy multiple skin niches, potentially increasing transmission risk within long-term care settings. Given the clinical importance of Enterobacterales, we next performed strain-resolved analyses of *E. coli* colonization dynamics.

### Metagenomics reveals dominant MDR *E. coli* ST93 missed by culture

**Fig. 3:**
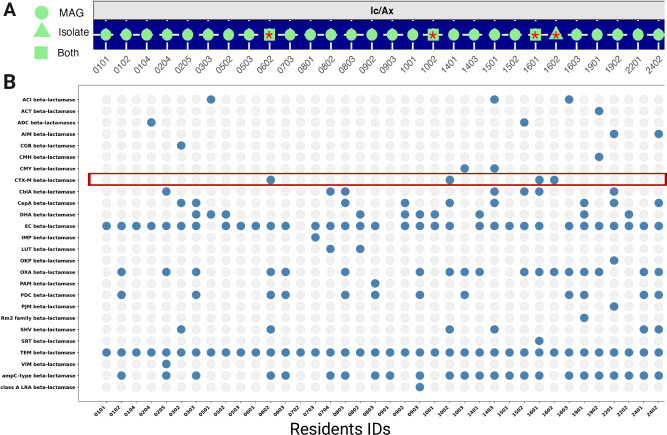
Nursing home residents’ skin colonization with multidrug resistant *E. coli.* **A** Detection of *E. coli* across residents with metagenome-assembled genomes (MAGs; circles), cultured isolates from ESBL-selective media (triangles), or both (squares). The red asterisk marks the β-lactamase gene content (i.e., carried *bla*_*CTX-M-15*_ and *bla*_*OXA-1*_.) of cultured *E. coli* isolates from ESBL-positive residents. **B** Presence/absence matrix of β-lactamase gene families recovered from metagenomic sequencing across all residents. Detection of *bla*_*CTX-M*_ genes (highlighted in red box) was confined to residents with cultured ESBL-producing isolates. In contrast, *E. coli* ST93 MAGs lacked *bla*_*CTX-M*_ but encoded other β-lactamase family genes and multidrug resistance determinants (see Supplementary Fig. 3). Figure panels were assembled using Biorender under publication license, Segre, J. (2026) https://BioRender.com/vgvb49x.

Genomic analyses of matched bacterial isolates and MAGs derived from shotgun metagenomic sequencing elucidated the *E. coli* strain that colonized resident skin. ESBL-selective culturing recovered *E. coli* from 4 residents (Fig. 3A), with genomic sequence analysis further delineating that 3 of these residents were colonized by *E. coli* ST131 and 1 resident was colonized by *E. coli* ST648. These four *E. coli* genomes carried multiple antibiotic resistance genes including *bla*_CTX-M-15_ and *bla*_OXA-1_ genes, which are known to confer an ESBL phenotype (Red asterisk in Fig. 3A; Supplementary Data 2). By contrast, MAG assembly from the skin metagenomic samples recovered *E. coli* ST131 MAGs from 4 residents, *E. coli* ST93 MAGs from 19 residents and additional singleton sequence types from 4 residents (Fig. 3A and Supplementary Data 3). None of the *E. coli* ST93 MAGs carried *bla*_CTX-M_ genes, nor was the *bla*_CTX-M_ gene detected in the corresponding skin metagenomes (Supplementary Data 4), providing a likely explanation of why these organisms did not grow on ESBL selective plates (Fig. 3B, red box). However, the *E. coli* ST93 MAGs still carried a broad MDR repertoire such as *bla*_TEM,_ AmpC-family β-lactamases, aminoglycoside, fluoroquinolone, macrolide, sulfonamide, and tetracycline resistance genes (in the absence of other Proteobacteria on the contigs as confirmed by strainGE and BLASTn) (Fig. 3B; Supplementary Fig. 3; Supplementary Data 4). Moreover, an *E. coli* ST93 isolate was recovered in the wider PROTECT study from a California nursing home resident (not in our microbiome cohort) carrying *bla*_CTX-M-1_ on an *Inc*I1 plasmid, demonstrating the lineage’s genetic capacity to acquire ESBL determinants. Together, these data reveal a two-tiered colonization landscape: low-prevalence ESBL-producing *E. coli* ST131 detected by cultivation and metagenomics and more prevalent MDR *E. coli* ST93 detected via metagenomics (Fig. 3).

### Regional clonal transmission of *E. coli* ST93 and diverse ST131 introductions

**Fig. 4:**
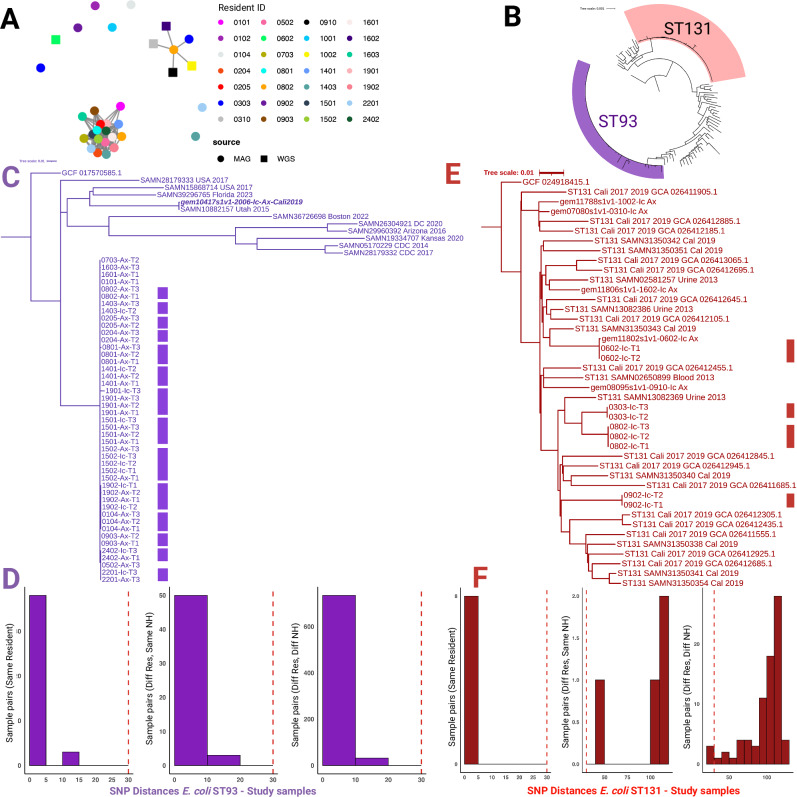
Comparative genomic analysis of *E. coli* strains from NH resident skin. **A** Network analysis of pairwise average nucleotide identity (ANI) among *E. coli* MAGs and isolate genomes from this study and the four isolate genomes from outside the cohort. Each node represents the highest-quality *E. coli* genome per resident, colored by resident. Circles denote MAGs and squares denote isolate whole genomes sequence (wgs). Edges connect genomes with ≥99.95% ANI, indicating high genetic relatedness. **B** Phylogenetic tree based on single-copy marker genes, including *E. coli* isolate genomes, MAGs from this study, and public reference genomes. Clades corresponding to *E. coli* ST131 and ST93 are highlighted in red and purple, respectively. **C** Phylogenetic tree of *E. coli* ST93 genomes from this study alongside representative references, inferred from core SNPs. The purple blocks along the tree mark samples from the same resident. gem10417s1v1_2006_Ic-Ax is the ESBL-producing *E. coli* ST93 obtained in another California NH in 2019. MAGs are labeled with residentID_bodysite_timepoint; **D** Histogram of pairwise core genome SNP distances among study-derived *E. coli* ST93 genomes. The red dashed line marks a 30-SNP threshold for strain-level relatedness. **E** Phylogenetic tree of *E. coli* ST131 genomes from study samples and public references, inferred from core SNPs. The red blocks along the tree mark samples from the same resident. **F** Histogram of pairwise core genome SNP distances between study-derived *E. coli* ST131 genomes and public *E. coli* ST131 references. The red dashed line denotes a 30-SNP threshold used to infer potential strain sharing. Abbreviations: Diff Subj different resident, Diff Fac different facilities/NHs. Figure panels were assembled using Biorender under publication license Segre, J. (2026) https://BioRender.com/s82td3a.

Consistent with sequence typing, a combined phylogenetic analysis of *E. coli* MAGs and isolate genomes (Fig. 4A, B) revealed two distinct clusters (ST93, ST131). A high-resolution SNP-based phylogeny of *E. coli* ST93 genomes showed a single clade circulating regionally across 15 NHs (Fig. 4C), with ≤30 SNPs between any pair of MAGs and ≤5 SNPs between MAGs from different timepoints or body sites of the same individual (Fig. 4D and Supplementary Data 5). Notably, 27 residents (71.1%) across 9 facilities shared clonally related *E. coli* ST93 strains (≤10 SNPs), suggesting widespread transmission or common-source exposure. The clonal, regional strains from this study were however >100 SNPs away from ESBL-producing *E. coli* ST93 obtained in another California NH in 2019, as well as other *E. coli* ST93 genomes from elsewhere in the country (Fig. 4C).

In contrast, *E. coli* ST131 genomes displayed greater diversity. While longitudinal samples within individuals were clonal (0–1 SNPs; Supplementary Data 6), intra-facility comparisons between residents typically exceeded 40 SNPs, suggesting multiple introductions. A few inter-facility comparisons (e.g., residents 0303 and 0802) approached potential transmission thresholds (28–52 SNPs) (Fig. 4E, F). Comparisons with public genomes from California highlighted occasional proximity to clinical *E. coli* ST131 isolates from California, implying regional overlap but overall high diversity. These results revealed a dominant, clonally conserved *E. coli* ST93 lineage spreading widely, contrasted with sporadic, diverse *E. coli* ST131 introductions.

### Methicillin-resistant *S. epidermidis* ST2 persists on skin and spreads across facilities

**Fig. 5:**
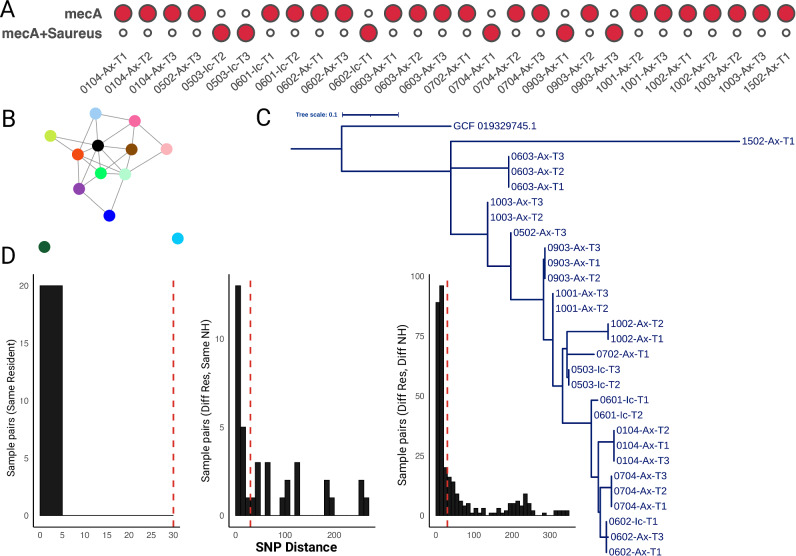
Persistence and resident strain sharing of methicillin resistant *Staphylococcus epidermidis* ST2 within and between nursing homes. **A** Detection of the *mecA* gene across metagenomic samples. Each circle represents a metagenomic sample. Red-filled circles indicate detection of *mecA*; *mecA* + *S. aureus* is indicative of co-presence by *S. aureus* in the metagenomic reads as identified by StrainGE. **B** Average nucleotide identity (ANI) network of *S. epidermidis* ST2 MAGs. Nodes represent individual *S. epidermidis* ST2 genomes, colored by resident, and edges connect genomes sharing ≥99.95% ANI. The clustering of genomes from different residents and facilities reflects the circulation of a highly conserved *S. epidermidis* ST2 lineage across the nursing homes. **C** Maximum-likelihood phylogenetic tree of *S. epidermidis* ST2 rooted to the *S. epidermidis* type strain (GCF_001922515.1). **D** Histogram of pairwise SNP-based genomic distance between *S. epidermidis* ST2 samples. Red dashed lines indicate the 30 SNP threshold for defining closely related strains. Figure panels were assembled using Biorender under publication license, Segre, J. (2026) https://BioRender.com/30fcvv9.

To determine whether transmission patterns extended beyond Enterobacterales, we next explored the prevalent methicillin-resistant *Staphylococcus epidermidis* (MRSE) ST2 in nursing home residents. *S. epidermidis* MAGs were detected in the skin microbiome of 84% of residents, with ST2 MRSE MAGs found in 14 residents (36.8%) (Fig. 5A). The *mec*A gene was detected in 23 of 28 *S. epidermidis* ST2 MAGs as well as in the remaining 5 samples co-colonized with *S. aureus*. Within-individual SNP distances ranged from 0 to 2, indicating clonal persistence. Average nucleotide identity (ANI) and SNP-based networks revealed closely related *S. epidermidis* ST2 strains circulating within and across facilities (Fig. 5B–D). Among the many examples, highly related strains (4–6 SNPs) were shared by residents 0601 and 0602 of the same NH, strains from resident 0903 differed by 3 SNPs from those of resident 0502, as well as related strains (41–43 SNPs) in residents 1001 and 1002 who are also in the same NH. Strains from different NHs were also highly related (e.g., residents 0503 and 0601 shared strains differing by 4 SNPs, 0104 and 0602 shared strains with only a maximum of 10 SNPs difference; 0704 and 0602 with 13–14 SNPs; 0903 and 0602 with 6 SNPs, 0104 and 0704 with 17 SNPs), suggesting regional dissemination of a predominant MRSE ST2 clone persisting on skin and potentially spreading between nursing homes.

### Proteus, enterococcus and providencia exhibit persistence and facility-level spread

**Fig. 6:**
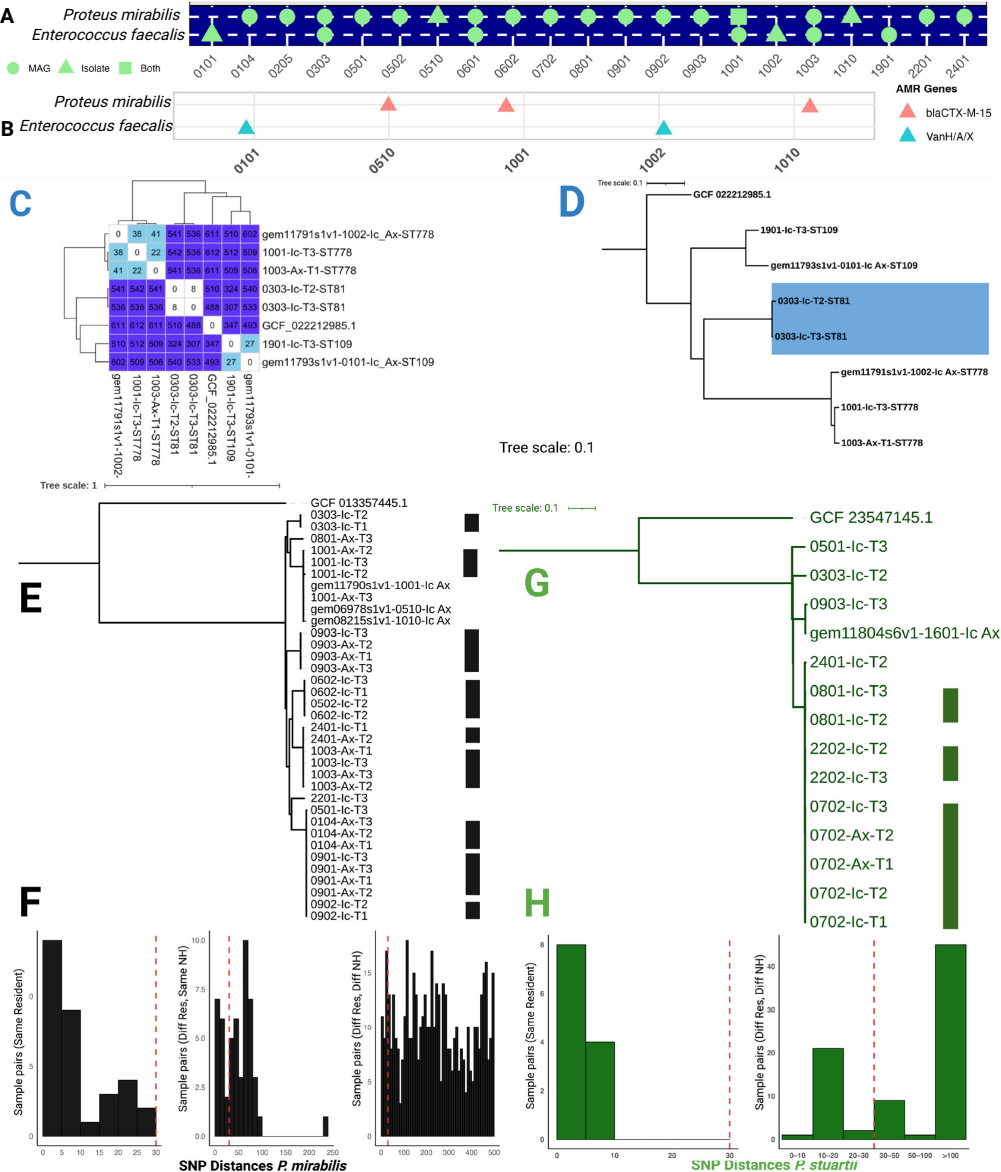
Genomic relatedness of healthcare-associated microbes colonizing nursing home residents. **A** Detection of *Proteus mirabilis* and *Enterococcus faecalis* on skin across NH residents, based on recovery as metagenome-assembled genome (MAG) from shotgun metagenomics, cultured isolate, or both. **B** Antimicrobial resistance gene detection in isolate genomes. *bla*_*CTX-M-15*_ was found in *P. mirabilis* isolates, while *vanHAX* operon genes (*vanH*, *vanA*, *vanX*) were identified in *E. faecalis*. Resident 0510 was not a participant in our skin microbiome sequencing cohort but resides in one of the NHs included in this study. **C** Pairwise SNP distance heatmap among *E. faecalis* strains. Lower values indicate greater genomic similarity. Samples starting with the identifier “gem” are isolate genomes. MAGs are labeled with ResidentID-bodysite-timepoint-ST. Light blue shading is 10-100 SNPs; Blue shading is 100–1000 SNPs. **D** Maximum likelihood phylogeny of *E. faecalis* isolates and MAGs, displaying clustering by resident and timepoint; the blue shade marks *E. faecalis* from the same resident. **E** Phylogenetic tree of *P. mirabilis* strains shows resident-specific clustering, highlighted with black side bars. **F** Histograms for *P. mirabilis* of SNP distances of genomes from the same resident, genomes of different residents within the same NH and genomes from residents of different nursing homes. **G** Phylogenetic tree of *Providencia stuartii* MAGs shows resident-specific clustering, highlighted with green side bars. **H** Histograms for *P. stuartii* comparing SNP distances of genomes from the same resident with between residents. Red dashed lines indicate the 30 SNP threshold for defining closely related strains. Figure panels were assembled using Biorender under publication license, Segre, J. (2026) https://BioRender.com/8nr8pf3.

Having established persistence and dissemination among the dominant lineages, we broadened the analysis to additional opportunistic pathogens detected on NH resident skin. *Proteus mirabilis* was detected in 18 residents (47.3%), including three ESBL-producing isolates carrying *bla*_CTX-M-15_ (Fig. 6A, B and Supplementary Data 2). *E. faecalis* was detected in 7 residents (18.4%), with VRE isolates typed as ST109 and ST778 (Supplementary Data 2). *E. faecalis* MAGs and isolates from 1001 and 1003 clustered phylogenetically (22–41 SNPs), consistent with NH-associated circulation, while the relatedness of a MAG from 1901 and an isolate from 0101 with 27 SNPs pointed to a possible regional reservoir (Fig. 6C, D). For *P. mirabilis*, SNP distances of 0–7 were observed among genomes recovered from the same resident, from residents within the same facilities and from residents in different facilities (e.g., 0104-Ax identical to 0901-Ax and 0902-Ic; 1003-Ax/Ic identical to 2401-Ax/Ic) (Fig. 6E, F and Supplementary Data 7). Extending this analysis to another species for which we recovered isolates and MAGs, *Providencia stuartii* was detected in 7 residents (18.4%), showing clonal persistence within individuals and near-identical strains (≤13 SNPs) shared between facilities (Fig. 6G, H; Supplementary Data 8). Additional MDROs included *P. aeruginosa* and *M. morganii* (Supplementary Fig. 4), often showing within-host clonality and occasional inter-resident sharing (<30 SNPs), while *K. pneumoniae* (ST20, ST1842) and *S. pettenkoferi* colonization remained largely resident-specific (Supplementary Figs. 5 and 6).

### Polymicrobial strain sharing reveals complex transmission networks

**Fig. 7:**
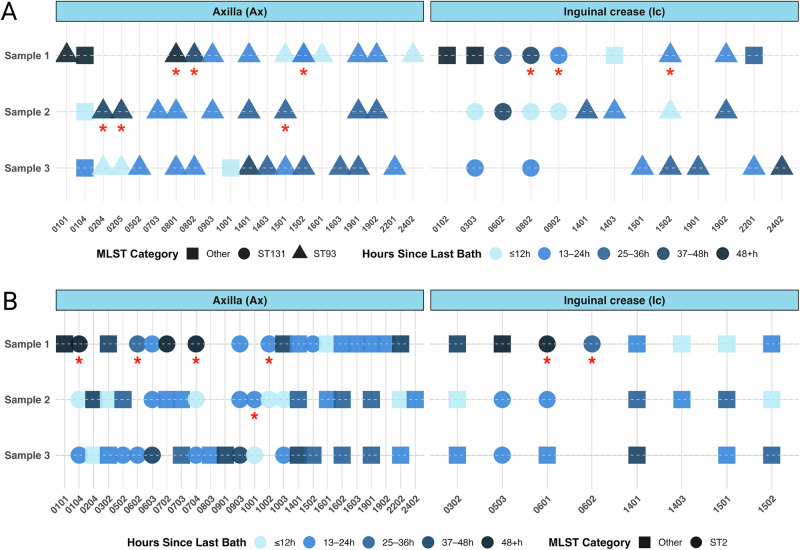
Persistent colonization of NH residents’ skin post-bathing. **A** Persistent recovery of *E. coli* ST131 and ST93 MAGs before and after bathing, stratified by body site (axilla [Ax] and inguinal crease [Ic]) and organized by resident. Shape represents STs, with the color indicating time since last bath ranging from 12 h (lighter) to 48 h (darker). The x-axis lists NH residents, and the *y*-axis indicates sampling events. Red asterisk (*) indicates when bathing occurred between sampling and is only applied for ST131 and ST93. Gaps represent no *E. coli* MAG detected. **B** Persistent recovery of *S. epidermidis* ST2 MAGs before and after bathing, stratified by body site (axilla [Ax] and inguinal crease [Ic]) and organized by resident. Each tile represents a sample; colored circles indicate recovery of an *S. epidermidis* ST2 MAG and are colored by time since last bath. Red asterisks mark persistent detection of *S. epidermidis* ST2 across multiple timepoints in the same resident. In the facet “Inguinal Crease” for resident 0602 there is an asterisk but no isolate indicated for Sample 2 or 3 because the individual had a bath and a subsequent MAG but this MAG did not meet the genomic criterion for completeness. Figure panels were assembled using Biorender under publication license, Segre, J. (2026) https://BioRender.com/eo9rqi6.

To integrate findings at the resident level, we aggregated our findings to explore possible polymicrobial strain sharing among nursing home residents. Five resident pairs harbored clonally related strains from ≥2 MDRO species, underscoring transmission networks. For example, residents 0902 and 0903 shared near-identical *P. mirabilis* (3–4 SNPs) and *E. coli* ST93 (1–3 SNPs) in NH 09 (Fig. 7). Cross-facility polymicrobial sharing was observed between NHs 08 and 22 (*P. stuartii*, *M. morganii*, ≤30 SNPs). Overall, 27 residents across 9 NHs (71.1%) shared at least one clonally related MDRO strain with another resident (≤10 SNPs), highlighting extensive intra- and inter-facility dissemination of multidrug-resistant skin colonizers.

### *E. coli* ST93 and ST131 persist on skin after bathing

As bathing was documented for this study, we leveraged our sampling on multiple days to assess whether *E. coli* strains identified by near-complete MAGs persisted on nursing home residents’ skin after bathing. Post bathing, persistent detection was observed in half the residents who were colonized by *E. coli*, including residents 0204, 0205, 0801, 0802, 1501, and 1502 carrying *E. coli* ST93, and 0802 and 0902 carrying *E. coli* ST131 (Fig. 7A). In every case, the same sequence type of *E. coli* was observed prior to and after the bath. For example, *E. coli* ST93 was detected on the axilla and inguinal crease of resident 1502 at both 12 and 24 h post-bath.

Resident 0802 is remarkable with *E. coli* ST93 persisting on the axilla and *E. coli* ST131 persisting on the inguinal crease after bathing (Fig. 7A).

Next, we sought to assess if there was a microbial community feature that distinguished residents with persistent skin colonization. Community-level microbiome profiles were broadly similar between persistent and non-persistent residents, indicating that post-bathing persistence occurred against a largely stable skin microbiome background. As described earlier, *E. coli* relative abundance was consistently higher in colonized compared to non-colonized samples across both body sites. In contrast, no clear differences in relative abundance were observed between *E. coli* persistent and non-persistent states. Alpha diversity at the pre-bathing timepoint did not differ between samples that remained colonized versus those that did not (Ax: *p* = 0.85; Ic: *p* = 0.53).

Similarly, persistent *S. epidermidis* ST2 colonization was observed over 12–48 h post-bathing in residents 1001, 1002, 0104, 0601, and 0602 (Fig. 7B; Supplementary Figs. 7–8, and Supplementary Data 9). As observed above, *S. epidermidis* relative abundance and skin microbiome alpha diversity did not differ between persistent and non-persistent sites. However, these analyses are limited by the co-occurrence of additional *S. epidermidis* strains within the same site.

## Discussion

AMR represents one of the greatest challenges to global health, with its burden projected to rise substantially in coming decades. Surveillance and response strategies have largely centered on acute-care hospitals and the livestock sector, leaving long-term care facilities, particularly nursing homes, underappreciated despite their role as persistent MDRO reservoirs^5,9^. Using a combined approach of shotgun metagenomics, MAGs, and isolate genome sequencing, we characterized MDRO colonization on nursing home residents’ skin and revealed ecological persistence, regional dissemination, and multi-species sharing events that are largely invisible to current culture- and peri-rectal-based diagnostics.

Our data demonstrate that MDRO colonization on skin is persistent rather than transient. Across multiple timepoints, clonal strains of *E. coli* (ST93 and ESBL-producing ST131), methicillin-resistant *S. epidermidis* (MRSE ST2), and vancomycin-resistant *E. faecalis* (ST109 and ST778) persisted despite bathing intervals of 12-36 h, consistent with previous findings that skin acts as a long-term reservoir for clinically relevant microbes capable of causing opportunistic infections^7^. Such persistence has implications for autoinoculation of wounds or medical devices, increased risk of infection, and ongoing environmental contamination that may sustain transmission cycles in NH settings.

One of the most striking findings was the widespread presence of *E. coli* ST93, detected in 71% of residents via MAGs yet absent from ESBL-selective culture plates. These strains lacked *bla*_CTX-M_ genes but carried a broad resistome, including *bla*_TEM_, AmpC β-lactamases, and resistance determinants for aminoglycosides, sulfonamides, and fluoroquinolones. The detection of an *E. coli* ST93 isolate in a neighboring nursing home carrying *bla*_CTX-M-1_ on a conjugative *Inc*I1 plasmid bordered by IS1380 elements provides direct evidence of this lineage’s potential to acquire ESBL genes, a configuration well documented in past ESBL epidemics^15,16^. Public data reveal additional high-risk variants of *E. coli* ST93, including isolates with *bla*_CTX-M-55_ and *bla*_NDM-1_ (SAMN28179333, SAMN39296765), as well as colistin resistance gene *mcr*−1 in Asia^17^, underscoring the lineage’s evolutionary potential to become a clinically significant MDRO. These results highlight that current resistance-targeted culturing may underestimate *E. coli* reservoirs in long-term care facilities, leaving silent dissemination unchecked.

Similarly, *S. epidermidis* ST2, a healthcare-adapted clone linked to device-associated infections in immunocompromised hosts^18,19^ was detected in 37% of residents, often shared clonally within and across facilities, with *S. epidermidis* ST2 MAGs differing by ≤10 SNPs. These strains frequently carried *mec*A and circulated regionally, yet none were captured by culturing since methicillin-resistant *S. epidermidis* was not specifically sought after during culturing. These findings suggest that dominant, clinically relevant lineages may be silently spreading within NHs, escaping detection by routine clinical workflows focused on hospital pathogens that rarely involve skin sampling.

Our metagenomic analysis further demonstrated that nursing homes act as interconnected nodes in regional transmission networks rather than isolated microbial ecosystems. Clonally related *E. coli* ST93, *E. faecalis* ST109/ST778, *Proteus mirabilis*, and *Providencia stuartii* strains were shared both within and between nursing homes, consistent with recent genomic epidemiology studies reporting MDRO dissemination among NH residents^9,20,21^. Frequent resident transfers between NHs and hospitals^5^, combined with limited infection prevention resources and delayed implementation of mandatory programs^22^, may contribute to this silent, inter-facility spread. Our data suggest that colonized residents, even in the absence of active infection, may serve as long-term, mobile reservoirs bridging care settings and contributing to regional MDRO circulation.

In several cases, we identified polymicrobial strain sharing where resident pairs carried clonally related strains from two or more distinct MDRO species. These events occurred both within facilities and across different nursing homes, involving combinations of *E. coli* ST93, *S. epidermidis* ST2, *E. faecalis*, *Providencia stuartii*, and *P. mirabilis*. These polymicrobial colonization patterns highlight that strain sharing patterns in nursing homes are not pathogen-specific but involve broader microbial consortia that may co-evolve, persist, and potentially exchange resistance genes on skin. Infection control strategies focused on single priority pathogens may therefore fail to interrupt the true ecological networks sustaining AMR in long-term care facilities. While our study did not integrate environmental samples like was done in other studies^8^, the use of shotgun metagenomics here gives a much wider overview on the constellation of culturable and non-culturable MDROs in the nursing homes.

Shotgun metagenomics provided critical insights into the skin resistome, detecting ARGs spanning multiple antibiotic classes, including β-lactams (*bla*_TEM_, *bla*_CTX-M_, *bla*_OXA_, *mec*A), aminoglycosides, sulfonamides, fluoroquinolones, phenicols, macrolides, and tetracyclines. Many of these genes were detected without corresponding culture isolates, highlighting the limitations of diagnostics dependent on phenotypic growth under selective conditions. For example, *E. coli* ST93 MAGs lacked ESBL determinants yet encoded multiple β-lactamases, illustrating a latent capacity for resistance that would remain undetected in standard screening. Although current binning algorithms limit direct plasmid-to-host ARG attribution^23,24^, read-level mapping confirmed key resistance determinants in high-risk lineages such as *E. coli* ST131 and ST93 (in the absence of other Proteobacteria on the contigs as confirmed by strainGE and BLASTn), supporting their potential clinical significance.

Collectively, the ecological and genomic data indicate that resistance-targeted culturing alone underestimates MDRO burden on skin. Integrating metagenomic surveillance alongside targeted culturing could close detection gaps and guide more effective infection-prevention strategies across long-term care and the healthcare networks they connect to.

This study has limitations. We sampled a maximum of three residents per nursing home, preventing detailed analysis of within-facility networks, including between roommates. Environmental sampling was not performed, restricting source tracking. Also, SNP-based thresholds (<30 SNPs) align with genomic epidemiology standards^9^ but cannot establish transmission directionality without denser temporal and epidemiological data.

Despite these limitations, our findings establish nursing home resident skin as a stable and overlooked reservoir of MDROs with potential for persistence and dissemination within and across facilities. The recurrent detection of polymicrobial strain sharing underscores that long-term care residents are exposed to overlapping reservoirs and transmission routes involving multiple resistant taxa. Culturing confirmed that these MDROs were viable and replicating on the skin while metagenomics enabled detection of additional pathogens of concern that were missed by selective culturing. Expanding surveillance frameworks to include skin as relevant site and nursing homes, employing genome-informed, multi-species strategies that integrate metagenomics with traditional culturing, could close critical detection gaps and guide more effective infection control. As the population of frail, immunocompromised individuals in long-term care grows, addressing colonization and transmissions within nursing homes will be crucial to curb the spread of AMR within healthcare ecosystems.

## Methods

### Study design, participants, and skin sample collection

This skin microbiome study was nested within Project PROTECT (NCT03118232), a cluster-randomized trial of 28 nursing homes in California, USA^5^. Between September and December 2018, a convenience sample of residents from facilities assigned to both routine care and decolonization arms were enrolled. A total of 38 residents from 15 NH were enrolled and contributed usable samples (Supplementary Data 1). Two skin sites (inguinal crease and axilla) were sampled at 2–3 visits per resident between 12 h and 48 h after bathing (Fig. 1A). These intertriginous sites provide robust and reproducible colonization signal and are well-suited for strain-resolved metagenomic investigation while reducing participant burden.

In total, we analyzed 223 samples, including 207 skin swabs, 6 sample controls (air swabs at sampling site), 6 negative controls (reagent-only controls), and 4 positive controls (Mock Community standard ZymoD6306, ZymoBIOMICS, Zymo Research, California, USA). The study was approved by the Institutional Review Board at the University of California, Irvine, and informed consent was obtained from all participants or their representatives.

### Culture-based microbiology

All 207 skin swabs and the 6 air swabs were plated on CHROMagar™ ESBL, CHROMagar™ VRE and CHROMagar™ MRSA to selectively isolate ESBL-producing Enterobacterales, vancomycin-resistant *Enterococcus* (VRE) and methicillin-Resistant *Staphylococcus aureus* (MRSA). Colonies were sub-cultured, and DNA extraction was performed using the QIAmp 96 DNA QIAcube HT kit or the Omega Bio-Tek Universal Pathogen Core Kit with modifications. Library preparation used Nextera XT kits, and sequencing (of 23 isolates excluding MRSA isolates) was performed on Ilumina NovaSeq X+ (Supplementary Data 2). Sequence reads were assembled with SPAdes^25^ v3.14.1, and quality assessed using QUAST^26^ v5.2.0 and CheckM2^27^ v1.0.2. Isolate genomes were analyzed for AMR genes with Abricate (using ResFinder v4.0 database^28^).

### Skin shotgun metagenomic sequencing and analysis

DNA from 207 skin swabs, 6 negative air swabs, 6 reagent-only controls, and 4 samples of the mock microbial community standard was extracted as previously described^10^. Nextera libraries were prepared and shotgun metagenomic sequencing was performed as paired-end reads (151 bp) on Illumina NovaSeq6000. Shotgun metagenomic reads were trimmed of adapters and low-quality bases with Cutadapt v4.0, and mapped to the T2T-CHM13v2.0 human reference genome using Bowtie2 v2.4.5 to remove host reads^29^. Quality metrics were assessed with FastQC and MultiQC.

MAGs were reconstructed using metaSPAdes v3.15.0 and MetaWRAP v1.2.2^30^, integrating MetaBAT2, MaxBin2, and CONCOCT for binning. MAGs were retained if completeness ≥50% and contamination ≤5% (CheckM2^27^ v1.0.2) as previously defined^10^. However, for strain sharing and persistence analyses near-complete MAGs were used (Median completeness of 97.45% and a median contamination of 0.45%). Chimeric assemblies were identified with GUNC v1.0.5^31^ and removed. Taxonomic assignment used GTDB-Tk (release 207^32^). Eukaryotic MAGs were screened with EukCC v2.1.1^33^ and classified using Mash v2.3^34^ and Mummer v3.23^35^ against fungal genome references (after downloading all fungal genomes in RefSeq on September 25th 2024). MAG assembly statistics, gene content metrics, and quality scores are provided in Supplementary Data 3.

Taxonomic composition of skin metagenomic samples was determined using Kraken2 v2.1.2^36^ with a custom database of the standard Refseq database (Refseq-Release226) enriched with dereplicated MAGs from this study. Abundance estimates were generated with Bracken v2.8.0 and imported into R (phyloseq v1.38). Antibiotic resistance genes (ARGs) were detected from the MAGs as well as from shotgun metagenomic reads by mapping with the resistance gene identifier (RGI) v5.2.1 integrated with the comprehensive antibiotic resistance database (CARD) v3.1.2^37^. When ARGs were not captured within near-complete MAGs, read-based detections were interpreted conservatively and evaluated in the context of strain-resolved MAG recovery. For these cases genes were considered present with ≥40% allele coverage and ≥70% nucleotide identity. To minimize potential cross-species misassignment of plasmid-borne resistance genes, ARG signals were conservatively attributed only in samples containing strain-resolved MAGs and lacking evidence of plausible alternative Proteobacteria species as assessed by StrainGE^38^ and BLASTn. Methicillin resistance was inferred by detection of mecA in *Staphylococcus epidermidis* MAGs in the absence of *S. aureus*.

### Strain-level analyses

Strain level analysis characterized MAGs and bacterial isolate genomes from this study, as well as dereplicated public genomes from RefSeq database for each species of interest downloaded on September 2nd, 2024. The genomic characterization of the ESBL Enterobacterales and VRE isolates recovered from NH residents and four comparative genomes from additional residents from the same facilities as part of the wider PROTECT trial^5^ are provided in Supplementary Data 2.

We performed Multi-locus Sequence Typing (MLST) for lineage assignment with mlst v2.23.0, using default settings and published pubMLST schema (https://pubmlst.org/, MLST Achtman). Phylogenomic analyses based on single-copy marker genes were built with GToTree^39^ and core-genome SNP based phylogenetic analysis for maximum likelihood trees with RaxML^40^ following previously described procedures^9^. Pairwise average nucleotide identity (ANI) was computed using FastANI v1.34.1^41^ to infer genomic similarity thresholds. Core-genome SNP profiling was performed with Snippy v4.4.1, with recombination masking using Gubbins v2.4.1^42^. SNP distances were calculated with snp-dists v0.8.2. Read-based strain detection from metagenomic sequences was performed using StrainGE v1.3.3^38^ with a database constructed with the combination of isolate genomes and dereplicated public genomes. A threshold of 30 SNPs was considered for phylogenetic relatedness based in the targeted species in NH settings from previous studies^9^.

### Statistics and reproducibility

This study is based on metagenomic and isolate genome sequencing analyses of skin swabs collected from nursing home residents enrolled in the Project PROTECT trial^5^. Sample sizes were determined by sample availability from the parent longitudinal cohort and no statistical method was used to predetermine sample size in this microbiome study. No data were excluded from the analyses other than standard microbiome filtering steps, where taxa present at <0.1% relative abundance or in <5% of samples were removed to reduce sparsity and sequencing noise. The experiments were not randomized, as this was an observational genomic analysis nested within an existing clinical trial cohort. Investigators were not blinded to sample allocation during downstream genomic and microbiome analyses.

All microbiome analyses were performed in R v4.3.2. Alpha and beta diversity metrics were computed from metagenomic profiles, and differences across body sites and time points were assessed by PERMANOVA. Pairwise SNP distances and phylogenetic relationships were reconstructed from whole-genome sequencing data and visualized using ggplot2 and iTOL^43^. Reported frequencies and percentages represent descriptive summaries of colonization prevalence and strain sharing patterns. Enrolled patient characteristics are shown in Supplementary Data 10.

## Supplementary information

Supplementary Information

Description of Additional Supplementary Files

Supplementary Data

Transparent Peer Review file

## Data Availability

Complete data availability information, including SRA accession numbers for metagenomic and isolate reads, is provided in Supplementary Data 1 and 2, respectively. The shotgun metagenomic reads are available in SRA under the BioProject ID: PRJNA1330355, while the sequence reads for isolate genomes were submitted to SRA under the BioProject PRJNA1301865.

## Code availability

Scripts and code used to generate the figures in this article are available at https://github.com/skinmicrobiome/2026_Hounmanou_NursingHome.
