# Supplementary material for: Strain sharing and persistence of microbial pathogens colonizing the skin of residents in a regional nursing home network
Source: Nat Commun. 2026 Jun 18;17:7703. doi: 10.1038/s41467-026-74611-x (PMC13434387; doi:10.1038/s41467-026-74611-x)
Supplement: Supplementary file 1 [file 41467_2026_74611_MOESM1_ESM.pdf]

Supplemental material for 'Strain sharing and persistence of microbial pathogens colonizing the skin of residents in a regional nursing home network' by Yaovi M.G. Hounmanou and colleagues.

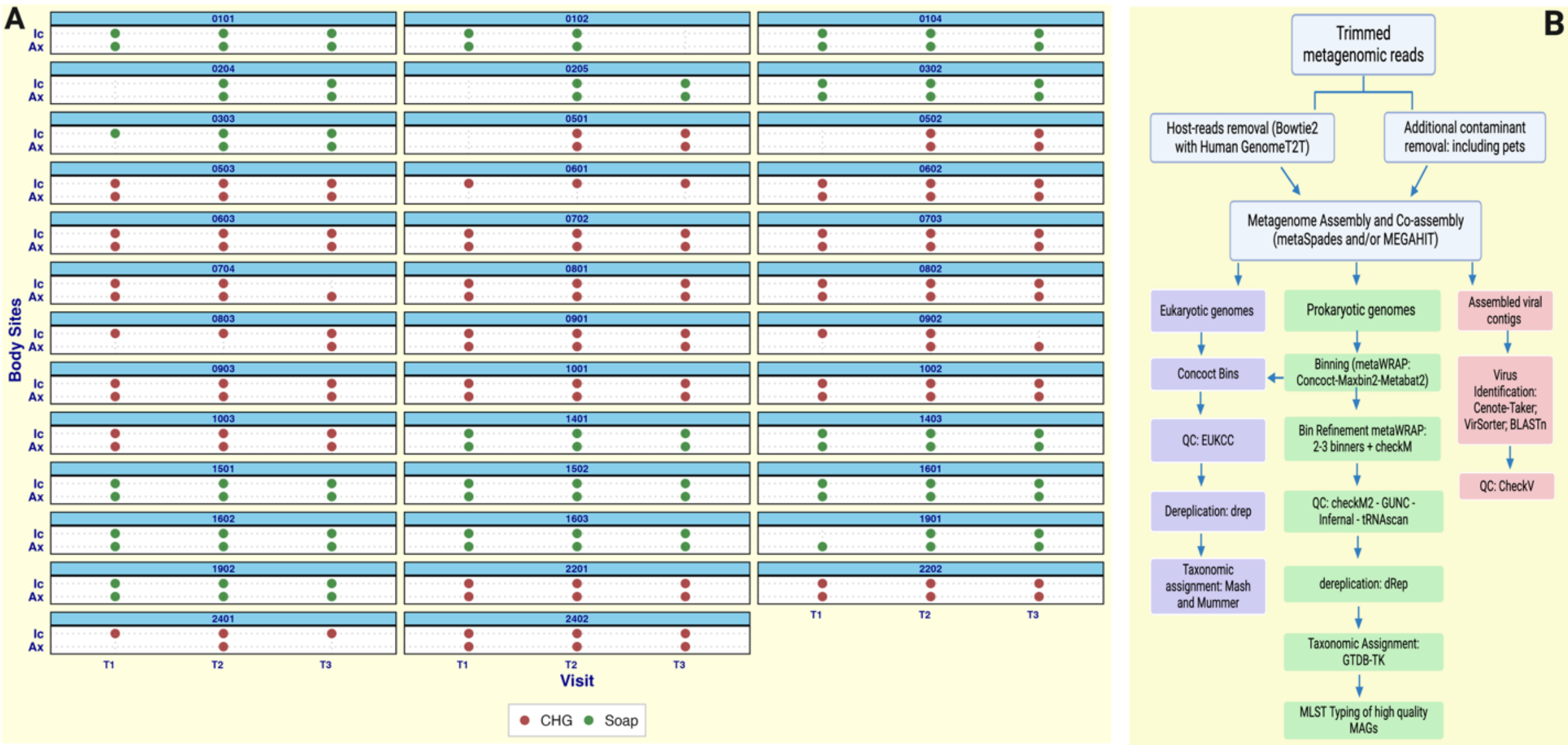

Supplementary Figure 1: (A) Overview of samples analyzed in this study and (B) overview of the analytical pipeline from sequences to MAGs. CHG = NHs where bathing is done with chlorhexidine gluconate. Soap = NHs where bathing is done with regular soap.

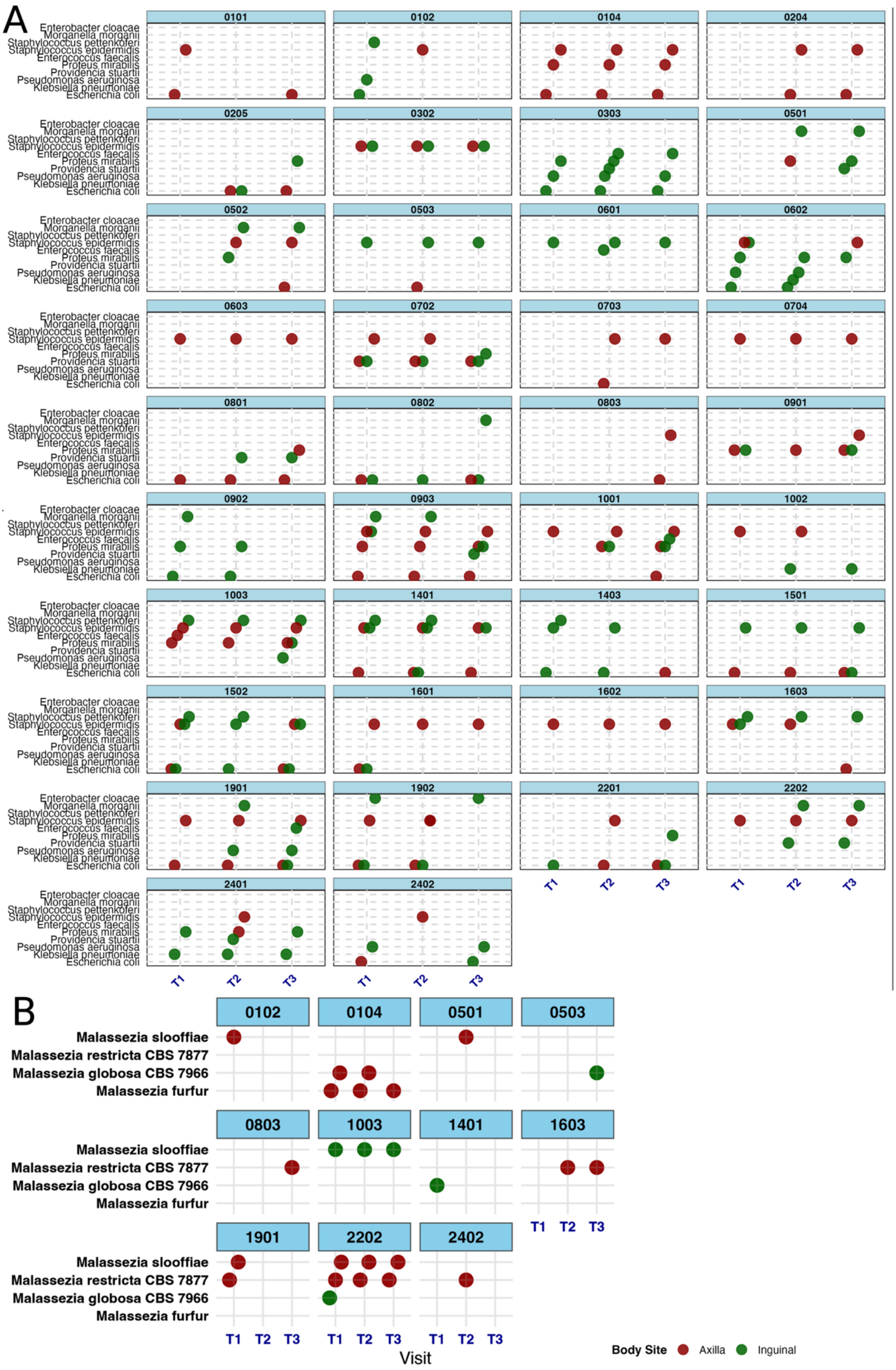

Supplementary Figure 2: Comprehensive overview of metagenome-assembled genomes (MAGs) from selected bacterial (A) and fungal (B) species of clinical relevance analyzed in this study.

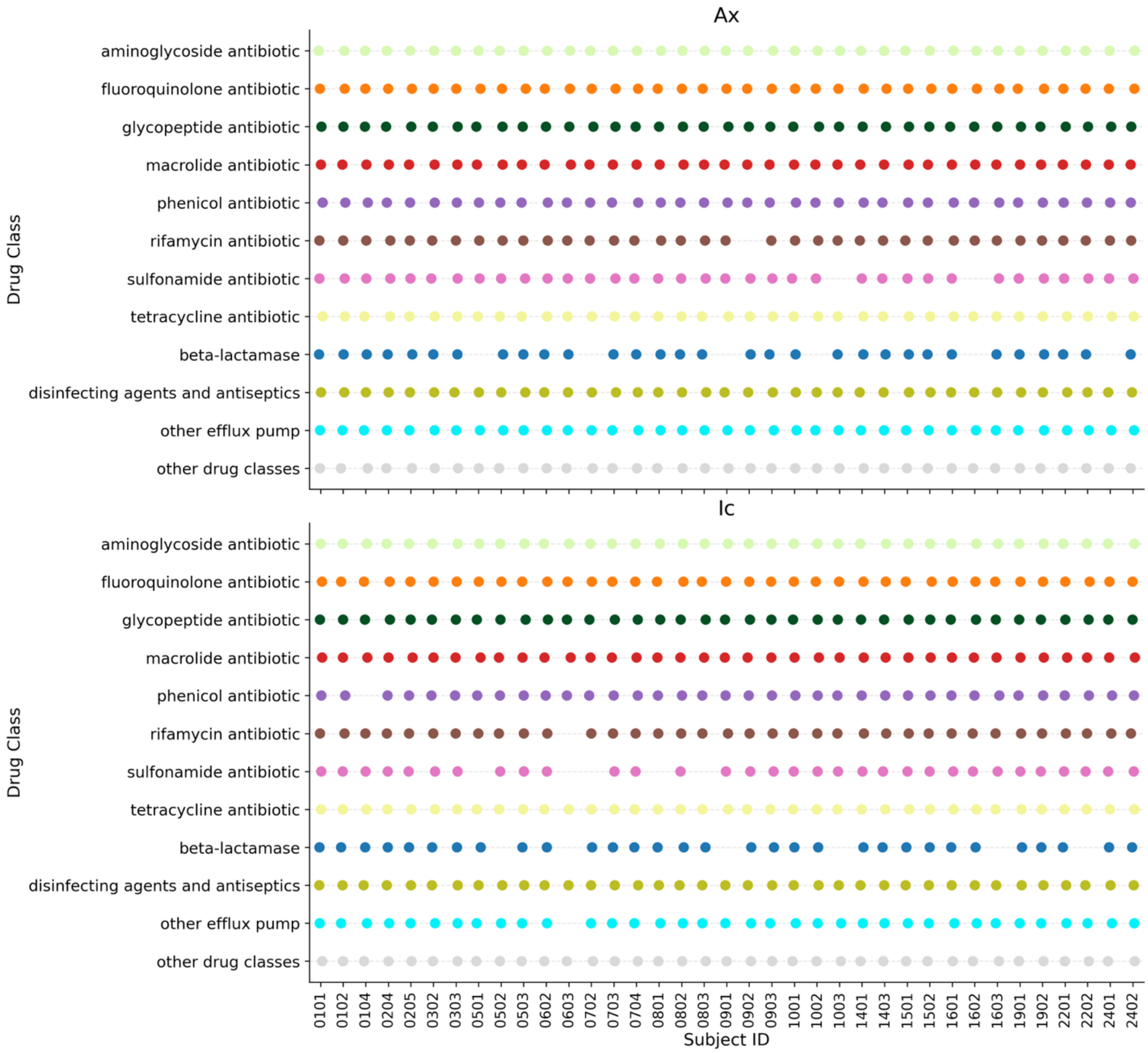

Supplementary Figure 3. Antibiotic resistance classes by resident across body sites. Dot plot showing the presence (colored dots) or absence (blank space) of major drug classes across individual residents, stratified by body site: axilla (Ax) and inguinal crease (Ic). Each row corresponds to a drug class, and each column to a resident ID.

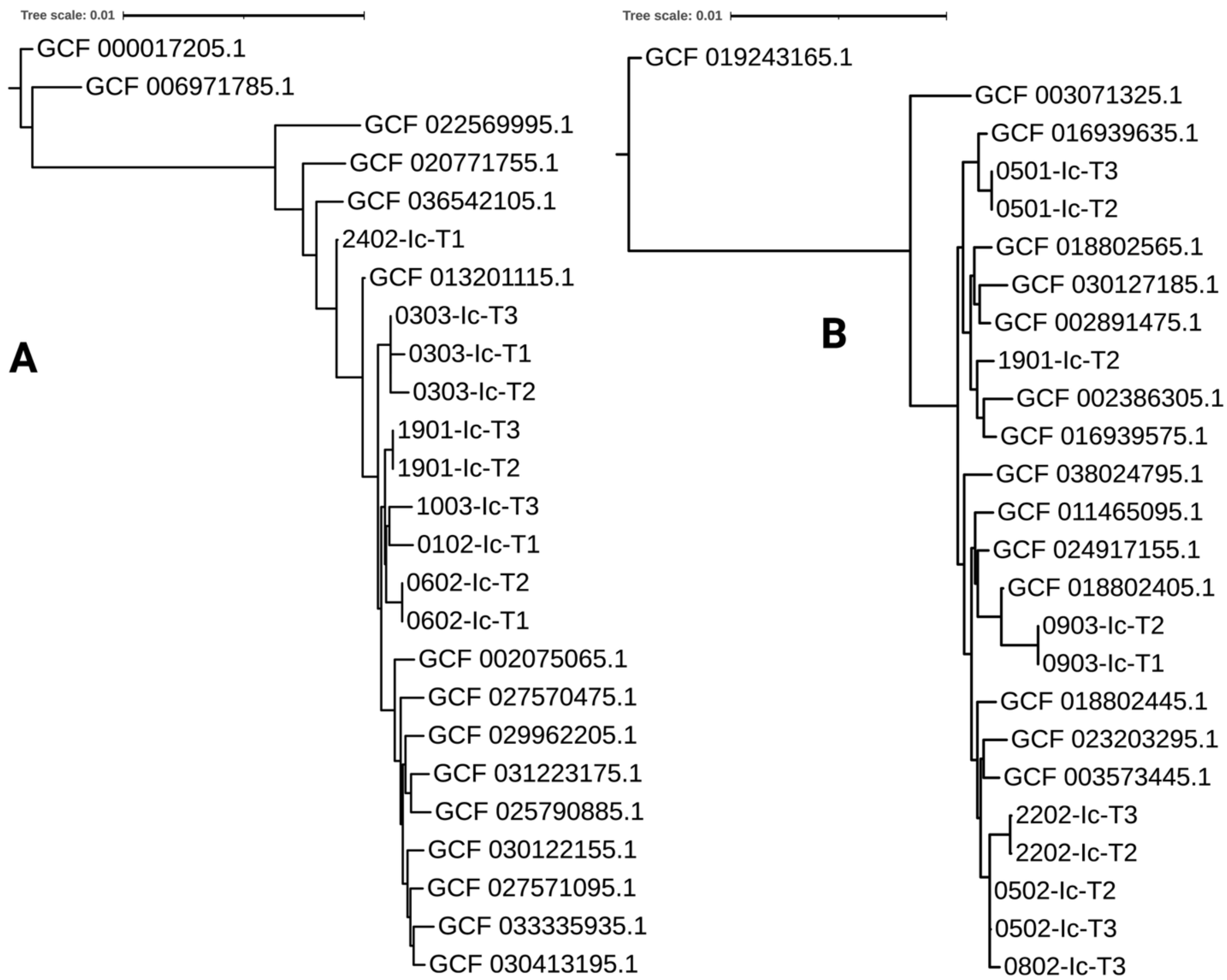

Supplementary Figure 4. Genomic relatedness of healthcare-associated microbes in nursing home Residents. (A) Phylogenetic tree of *Pseudomonas aeruginosa*. (B) Phylogenetic tree of *Morganella morganii*.

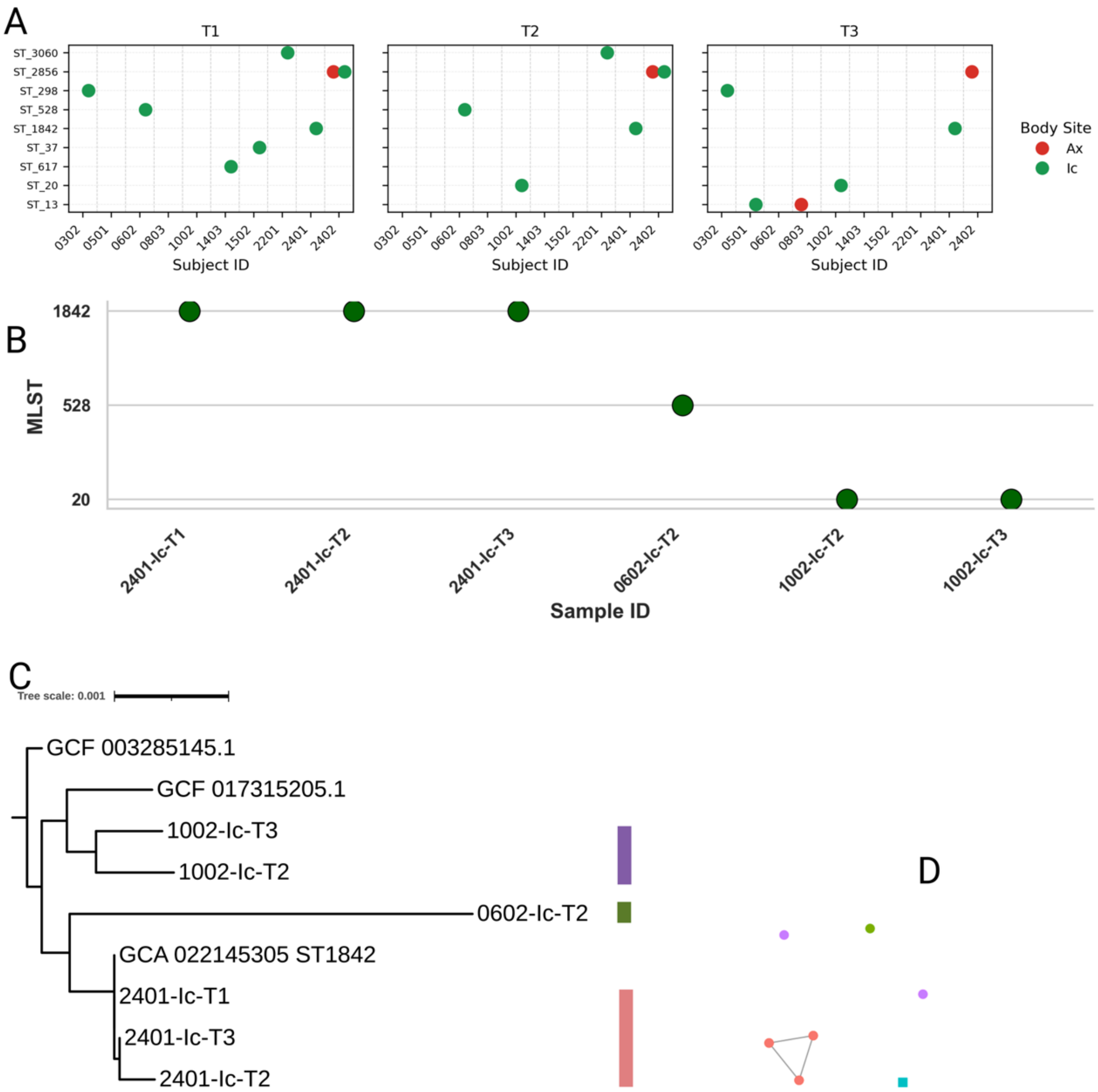

Supplementary Figure 5. Detection and genomic characterization of *Klebsiella pneumoniae* on NH resident skin. **(A)** Read-level detection of *K. pneumoniae* strains across three timepoints using StrainGE. **(B)** MAG-derived multilocus sequence typing (MLST) for samples with near-complete *K. pneumoniae* genomes. **(C)** Phylogenetic tree of *K. pneumoniae* MAGs from NH residents alongside reference genomes. **(D)** Average nucleotide identity (ANI) network confirming tight clustering of strains within individuals and separation between residents.

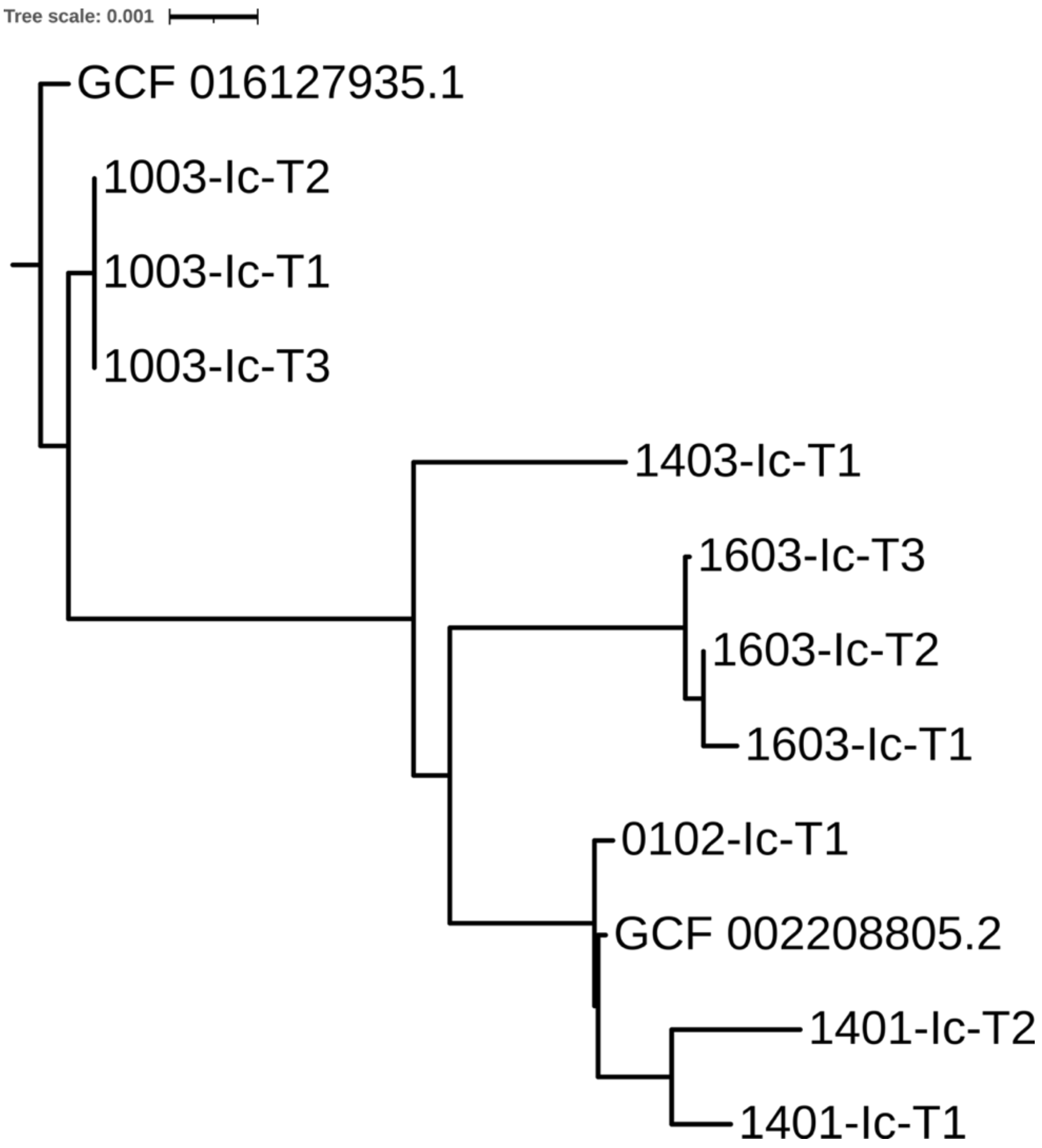

Supplementary Figure 6. Within-Host Persistence of *Staphylococcus pettenkoferi* Strains in Nursing Home Residents. Single copy marker gene phylogenetic tree of *S. pettenkoferi* MAGs.

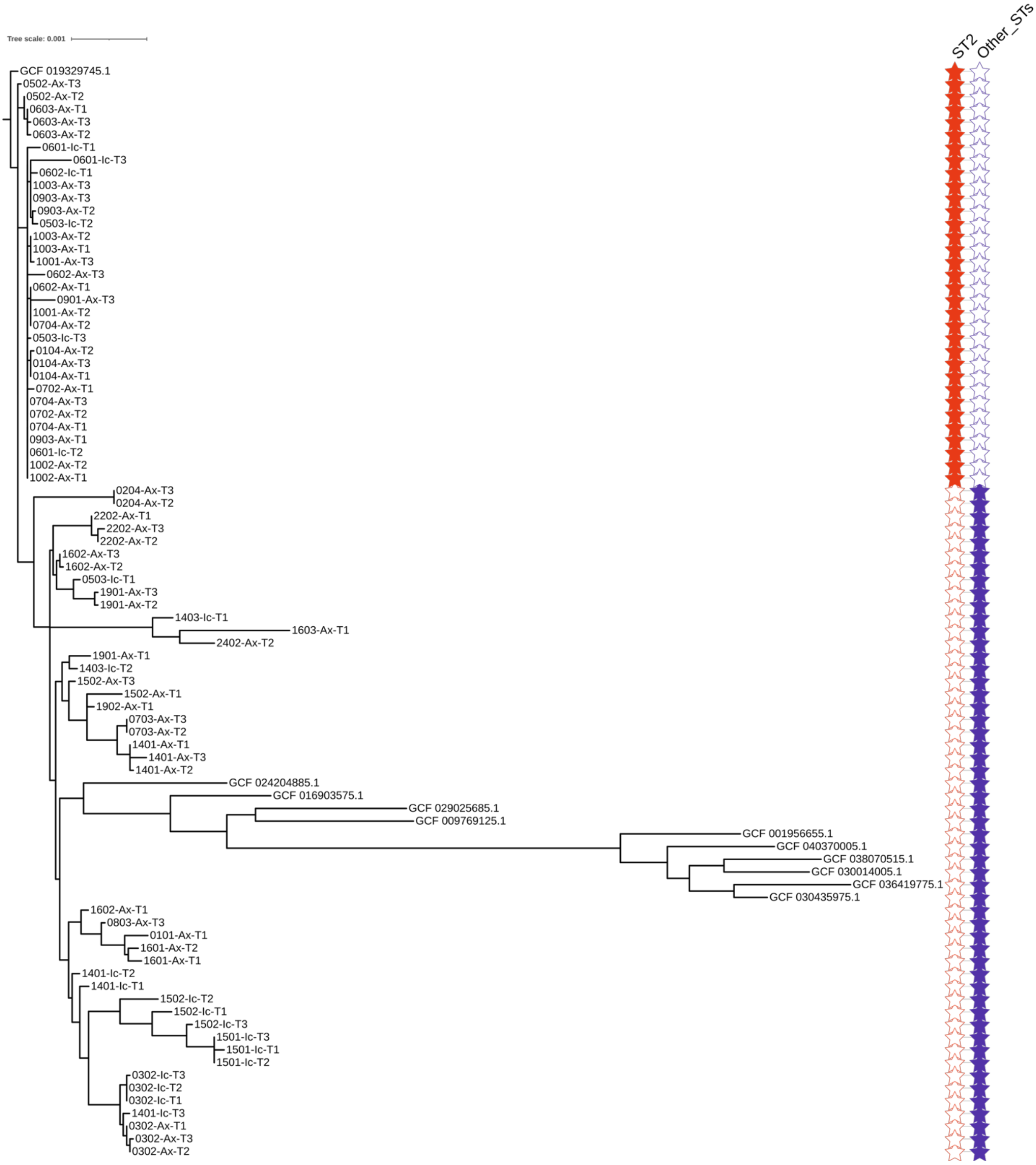

Supplementary Figure 7: Overview of single copy marker gene tree of all *Staphylococcus epidermidis* analyzed along with public genomes

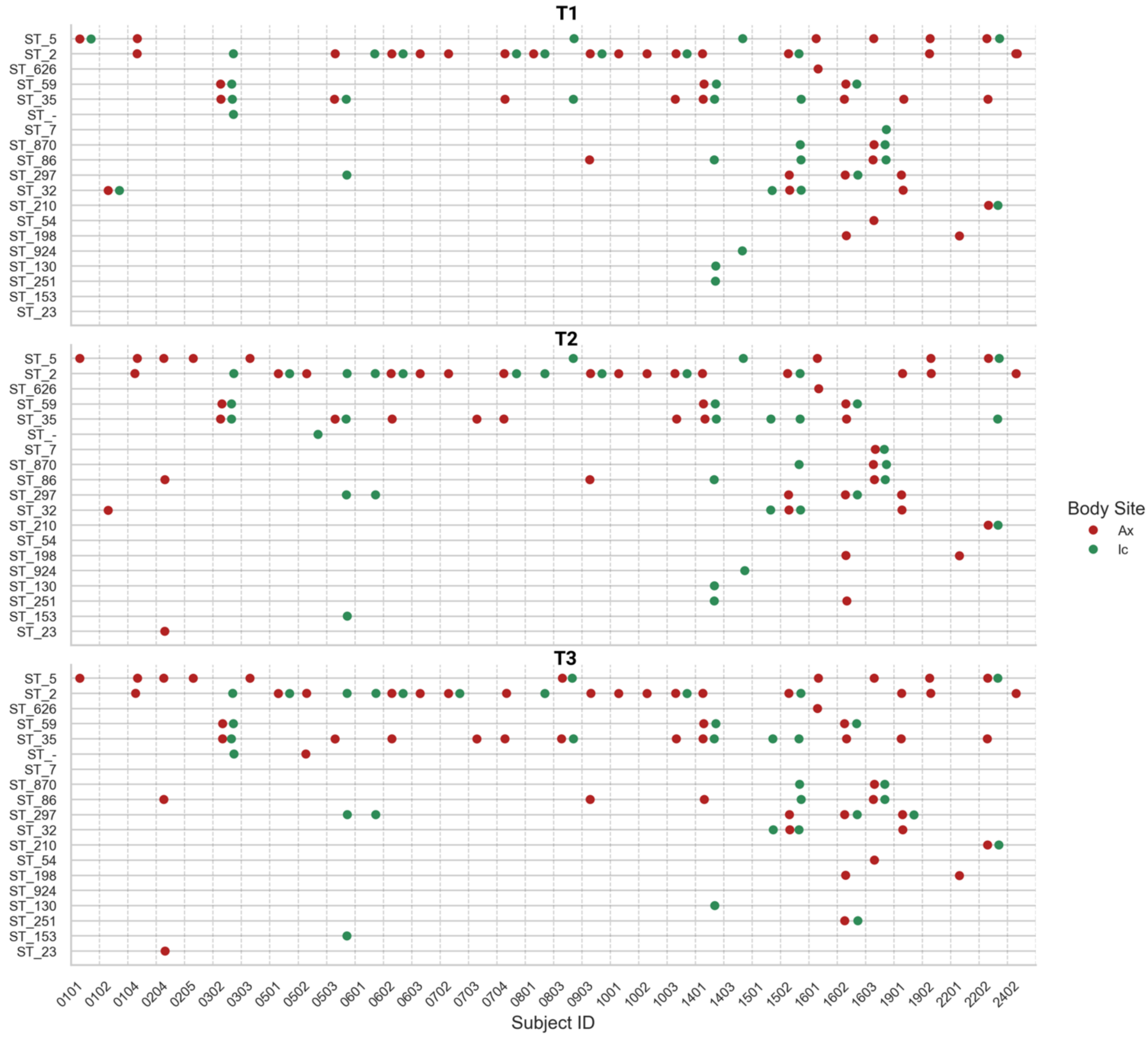

Supplementary Figure 8. Persistent detection of multiple sequence types of *Staphylococcus epidermidis* by StrainGE in shotgun metagenomic data.
