# Supplementary material for: Strain sharing and persistence of microbial pathogens colonizing the skin of residents in a regional nursing home network
Source: Nat Commun. 2026 Jun 18;17:7703. doi: 10.1038/s41467-026-74611-x (PMC13434387; doi:10.1038/s41467-026-74611-x)
Supplement: Supplementary file 2 [file 41467_2026_74611_MOESM2_ESM.pdf]

Supplementary Data for 'Strain sharing and persistence of microbial pathogens colonizing the skin of residents in a regional nursing home network' by Yaovi M.G. Hounmanou and colleagues.

SUPPLEMENTARY Data 1
Description: Shotgun metagenomic data from skin samples

SUPPLEMENTARY Data 2
Description: Isolate genome sample and sequence characteristics

SUPPLEMENTARY Data 3
Description: MAGs recovered with taxonomic and quality information

SUPPLEMENTARY Data 4
Description: Antibiotic resistance gene mapping of shotgun metagenomic samples

SUPPLEMENTARY Data 5
Description: SNP analysis for *E coli* ST93 MAGs and reference genomes

SUPPLEMENTARY Data 6
Description: SNP analysis for *E coli* ST131 isolates and MAGs

SUPPLEMENTARY Data 7
Description: SNP analysis for *S epidermidis* ST2 MAGs

SUPPLEMENTARY Data 8
Description: SNP analysis for *Providencia stuartii* isolates and MAGs

SUPPLEMENTARY Data 9
Description: SNP analysis for *Proteus mirabilis* isolates and MAGs

SUPPLEMENTARY Data 10
Description: Patient/resident characteristics
