# Supplementary material for: Strain sharing and persistence of microbial pathogens colonizing the skin of residents in a regional nursing home network
Source: Nat Commun. 2026 Jun 18;17:7703. doi: 10.1038/s41467-026-74611-x (PMC13434387; doi:10.1038/s41467-026-74611-x)
Supplement: Supplementary file 3 [file 41467_2026_74611_MOESM4_ESM.pdf]

nature portfolio

Peer Review File

Strain sharing and persistence of microbial pathogens colonizing the skin of residents in a regional nursing home network

Corresponding Author: Dr Julia Segre

This file contains all reviewer reports in order by version, followed by all author rebuttals in order by version.

Version 0:

Reviewer comments:

Reviewer #1

(Remarks to the Author)
The study by Hounmanou Y et al. investigates the persistence and transmission of the multi-drug-resistant bacterial pathogens on the skin of the residents in nursing home settings across California. The study employs both 1.) selective culturing followed by WGS and 2) Direct short gun metagenomics sampling from skin swabbing at axilla and groin. The longitudinal sampling identified persistent colonization and clonal transmission of E. coli ST93 and ST103, Methicillin-resistant S. epidermidis ST2, among the residents. Further, analysis of SNPs in core genes among the transmitted isolates for epidemiological tracing the strain shared among the nursing home residents. Overall, the study is well executed, and the study data is convincing. But the representation of the data is not systematic and may require a structure. Further, I have raised additional comments to improve the outcome of this current study.

1.) The manuscript requires structure in presenting the data. The current version is not structured, and it is difficult as a reader grasp the data. Most of the figures have same repetitive patterns of data presentation. If this can be rearranged and well structured, it will be easy for the readers to interpret the findings.

2.) In my opinion, the persistent colonization from longitudinal sampling (of E. coli, and S. epidermidis) after bathing can be in one separate figure.

3.) Is there any particular reason why only axilla and groin regions were sampled in this study? The study did not include skin sampling from other sites like anterior nares, palms, toe webs and perianal region.

4.) The longitudinal sampling revealed persistent E. coli ST93, ST103 and S. epidermidis ST2 among the residence. It may be meaningful to analyze the skin microbiome associated with the persistent colonization in these residents. Relative abundance, alpha diversity and beta diversity of microbiome from these resident’s samples may reveal interesting findings.

5.) The introduction of SNPs in the core genes suggests clonal expansion. It is worth to analyze if the clonally expanding E. coli 131 and S. epidermidis ST2 genome had harbored any additional accessory genes (related to anti-microbial resistance) over the spread among different residents and facility. This will add more value to this study.

6.) Also, do authors have antibiotic susceptibility profile of all these clonally expanded lineages of E. coli 131 and S. epidermidis ST2 among different resistance and different nursing home settings?

Reviewer #2

(Remarks to the Author)
Hounmanou et al combine microbial culturing, shotgun metagenomics and genome/metagenome assembly to show that multiple microbial strains and anti-microbial resistance genes are shared within and between nursing homes, with residents’ skin serving as a reservoir for acquisition and persistence. The study further illustrates the added value of metagenomic sequencing for detecting microbes of potential clinical concern in a vulnerable population that would be difficult to capture

comprehensively by culture-based approaches alone. Overall, the data support the main observations and merit publication, although several aspects of interpretation and presentation would benefit from clarification, as outlined below:

1. The authors posit “a two-tiered colonization landscape” of two E. coli strains: ST131(ESBL +; less prevalent) and ST93 (ESBL -; more prevalent). In rarer cases where the two strains co-exist e.g. on resident 0802, are the strain relative abundances estimated by StrainGE comparable, or does ST93 dominate the sample? By extension, are most axilla and inguinal crease samples dominated by a single lineage of E. coli? Do relative strain abundances of E. coli and species like S. epidermidis with high intra-species diversity change following bathing? These patterns would be informative for assessing whether intra-species competition limits co-colonization, as observed for other skin microbes (PMID: 40588591).

2. In several places (e.g., lines 174–176 and 191–194; Fig. 3 legends), the manuscript describes specific AMR genes as being carried by E. coli MAGs of specific strains. However, the methods indicate that these resistance determinants were detected by read-level mapping. Because many of these AMR genes are typically plasmid-borne and can circulate among multiple Proteobacteria, it is unclear how strainGE or BLASTn could confidently assign plasmid-derived contigs harboring these genes specifically to E. coli strains. This is especially relevant given that other Proteobacteria are present at these sites (Fig. 1B) and could plausibly serve as reservoirs for the detected AMR genes.

3. The authors make multiple references to “strain transmission dynamics” (e.g. lines 82 and 297) in the manuscript. However, aside from within-individual bathing comparisons, the analyses of inter-facility spread are cross-sectional and do not track the movement of specific strains between individuals or facilities over time. As such, it would be helpful to clarify whether the authors are referring to ‘ transmission dynamics’ in a temporal sense, or to adjust the terminology to better reflect the static nature of the available data for cross-site analysis.

4. Line 436: The authors write that “Culturing confirmed that these MDROs were viable and replicating on the skin…” This over-interprets the data, as most species and strains detected by metagenomics were not successfully cultured in this study. While microbial recovery post-bathing provides indirect support for viability, it could also reflect re-seeding from the environment. To strengthen claims about active replication, the authors could consider computational approaches such as iRep (PMCID: PMC5538567) or GriD (PMID: 30470746) to estimate growth rates of specific species from metagenomic data.

5. Line 283: “Asterix” should be asterisk.

6. Panels in figure 7 should be more clearly labelled as to which species are being analysed, because C-D, E-F and G-H relate to three different species.

7. Line 536: “Full computational workflows, detailed software parameters, command-line scripts and codes are provided on github.com/skinmicrobiome.” For clarity, please change the link to specific github repository for this project.

Reviewer #3

(Remarks to the Author)
I co-reviewed this manuscript with one of the reviewers who provided the listed reports. This is part of the Nature Communications initiative to facilitate training in peer review and to provide appropriate recognition for Early Career Researchers who co-review manuscripts.

Version 1:

Reviewer comments:

Reviewer #1

(Remarks to the Author)

The authors addressed the comments. no further concerns.

Reviewer #2

(Remarks to the Author)
The authors have fully addressed my concerns raised in the initial review, with appropriate revisions and additional analyses in the rebuttal document. I support the publication of the revised work.

Reviewer #3

(Remarks to the Author)
I co-reviewed this manuscript with one of the reviewers who provided the listed reports. This is part of the Nature Communications initiative to facilitate training in peer review and to provide appropriate recognition for Early Career Researchers who co-review manuscripts.

Open Access This Peer Review File is licensed under a Creative Commons Attribution 4.0 International License, which permits use, sharing, adaptation, distribution and reproduction in any medium or format, as long as you give appropriate credit to the original author(s) and the source, provide a link to the Creative Commons license, and indicate if changes were made.
In cases where reviewers are anonymous, credit should be given to 'Anonymous Referee' and the source.
The images or other third party material in this Peer Review File are included in the article's Creative Commons license, unless indicated otherwise in a credit line to the material. If material is not included in the article's Creative Commons license and your intended use is not permitted by statutory regulation or exceeds the permitted use, you will need to obtain permission directly from the copyright holder.
To view a copy of this license, visit https://creativecommons.org/licenses/by/4.0/

REVIEWER COMMENTS

Reviewer #1 (Remarks to the Author):

The study by Hounmanou Y et al. investigates the persistence and transmission of the multi-drug-resistant bacterial pathogens on the skin of the residents in nursing home settings across California. The study employs both 1.) selective culturing followed by WGS and 2) Direct short gun metagenomics sampling from skin swabbing at axilla and groin. The longitudinal sampling identified persistent colonization and clonal transmission of E. coli ST93 and ST103, Methicillin-resistant S. epidermidis ST2, among the residents. Further, analysis of SNPs in core genes among the transmitted isolates for epidemiological tracing the strain shared among the nursing home residents. Overall, the study is well executed, and the study data is convincing. But the representation of the data is not systematic and may require a structure. Further, I have raised additional comments to improve the outcome of this current study.

We thank the reviewer for their assessment of the strengths and weaknesses of the work, for their time in reviewing and for providing such constructive comments to improve the study. Your expertise in this subject, together with your specific suggestions have enhanced the lucidity of this manuscript. Our responses are found under each comment below as well as incorporated in the revised manuscript.

1.) The manuscript requires structure in presenting the data. The current version is not structured, and it is difficult as a reader grasp the data. Most of the figures have same repetitive patterns of data presentation. If this can be rearranged and well structured, it will be easy for the readers to interpret the findings.

We thank the reviewer for this important comment regarding the organization and presentation of the data. We acknowledge that there are two distinct ways to present these analyses - organized around either study datatype or bacterial species. We understand the reviewer’s suggestion for a more theme-based structure (e.g., grouping all persistence findings together across species). Such an approach has advantages, and we have tried to incorporate this perspective as much as possible. We have now restructured the manuscript to describe persistence more generally as suggested. However, we also found that this thematic presentation reduced some of the clinical interpretability which requires a species-specific context. So we open with an overall assessment of skin microbime; then we have kept the transmission tracking, strain-level analyses at the species level. Species specific analyses for strain tracking shows how our data would compare with current approaches in order to then tie the species-agnostic approach together in the end. As suggested by the reviewer we then pull together the theme of persistence to conclude the results section. [this topic of presenting persistence is further expanded by question 2 and 4 below]. Your suggestion greatly improved the lucidity of the manuscript to put forth genomics as a global solution that rises above the species-specific current approach.

As well to improve readability, we added introductory text at the beginning of the Results section to explicitly guide the reader through the intended narrative and improved figure referencing and transitions between sections. We hope these revisions improve the clarity and overall structure of the manuscript while preserving the clinically meaningful organization of the findings.

2.) In my opinion, the persistent colonization from longitudinal sampling (of E. coli, and S. epidermidis) after bathing can be in one separate figure.

We thank the reviewer for this thoughtful suggestion, which helped to restructure the manuscript. The persistence of Gram-negative *E. coli* (not considered a skin commensal) and Gram-positive *S. epidermidis*

(skin commensal) are of distinct biological and epidemiological importance on skin. Our previous distinct presentations missed the opportunity to discuss the similarity and differences we found in our studies of these two different organisms. We have the persistence figures following your suggestion to combine E. coli and S. epidermidis. We hope that the newly structured manuscript improves clarity while maintaining biological coherence.

3.) Is there any particular reason why only axilla and groin regions were sampled in this study? The study did not include skin sampling from other sites like anterior nares, palms, toe webs and perianal region.
This is a very astute question. In line with our previous studies, the anterior nares were included in the initial sampling scheme; however, routine nasal iodophor as part of the decolonization practice in these nursing homes, resulted in markedly reduced microbial biomass in the nares of residents in this cohort. While we attempted to deeply sequence the nares samples with 30 million reads, the sequencing libraries were dominated by human DNA, limiting robust metagenomic profiling and reliable genome recovery. In most nasal samples, only ~100,000 microbial reads were recovered (see slide below), and inclusion of these low-biomass samples would therefore introduce systematic site-specific bias and potential underestimation of overall colonization. Nasal samples consequently did not meet prespecified quality thresholds and were failed/excluded from downstream analyses.

The focus on the axilla and inguinal crease was epidemiologically motivated. Prior multi-site nursing home studies from our group have demonstrated that these moist intertriginous sites are high-biomass, epidemiologically relevant skin reservoirs that reproducibly exhibit high MDRO burden with stable colonization hypothesized (PMID: 40011766; PMID: 34155414). Axilla/groin are also the CDC-recommended sites of skin surveillance for *C. auris, A. baumannii.* And as such we were following a typical clinical surveillance protocol under healthcare quality improvement. We have clarified this rationale in the revised Introduction and Methods.

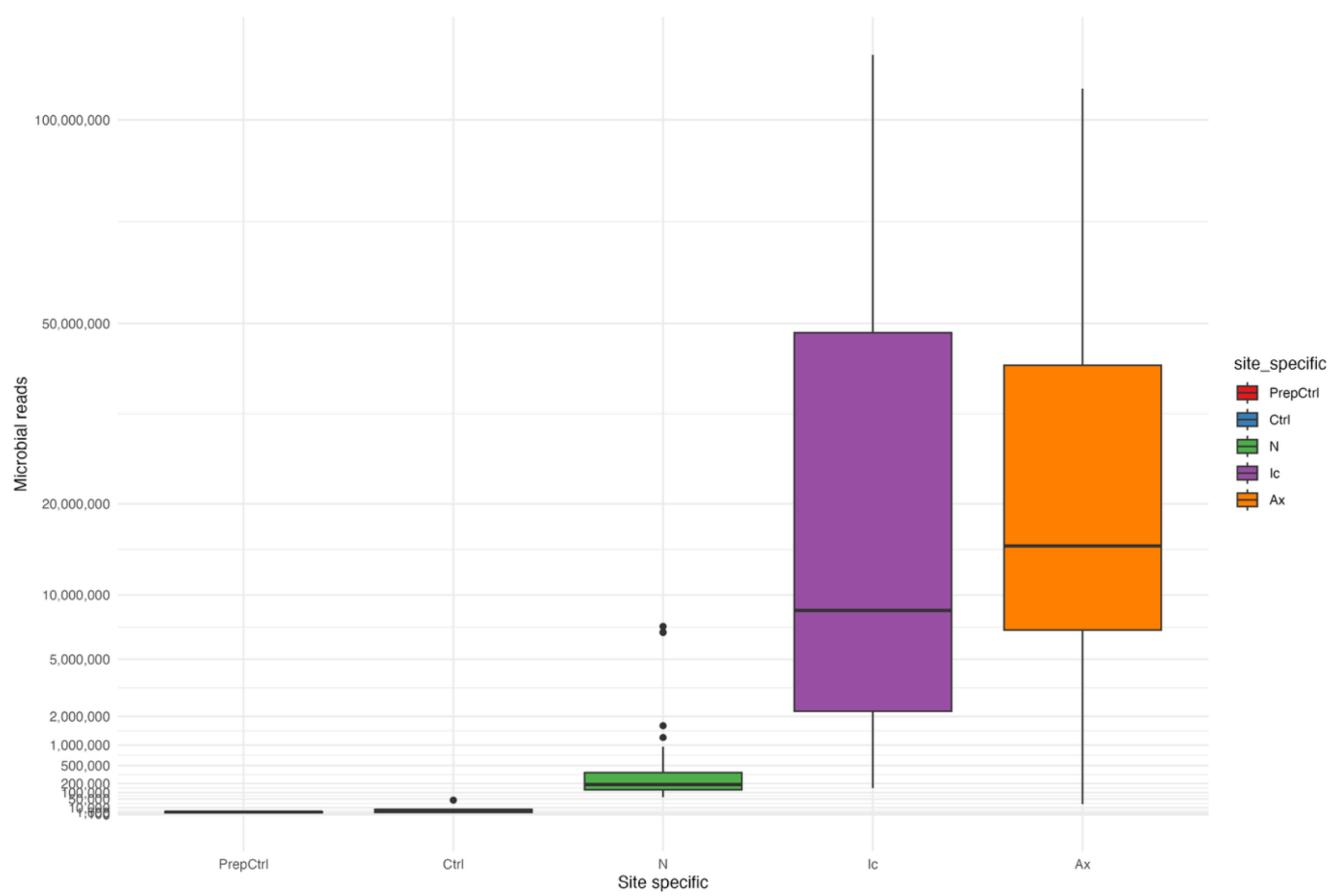

4.) The longitudinal sampling revealed persistent E. coli ST93, ST103 and S. epidermidis ST2 among the residence. It may be meaningful to analyze the skin microbiome associated with the persistent colonization in these residents. Relative abundance, alpha diversity and beta diversity of microbiome from these resident's samples may reveal interesting findings.

To address the reviewer's question, we first defined colonization status using an independent strain-resolved approach. Specifically, StrainGE outputs were used to detect *E. coli* ST93 and ST131 based on genome coverage, applying a conservative threshold (≥0.4) to define confident colonization at each sampling point. Persistence was then defined longitudinally by comparing colonization status across consecutive samples from the same individual and body site, classifying observations as "persistent" (concordant detection) or "non-persistent" (discordant detection). Using these strain-informed labels, we evaluated community-level patterns in the phyloseq-derived microbiome dataset, including *E. coli* relative abundance, alpha diversity (Shannon index), and beta diversity (Bray-Curtis dissimilarity), with appropriate statistical testing (Wilcoxon tests and PERMANOVA with dispersion assessment).

At the species level, *E. coli* relative abundance was consistently higher in colonized compared to non-colonized samples across both body sites, confirming concordance between strain-level detection and species-level abundance patterns. In contrast, no clear differences in relative abundance were observed between persistent and non-persistent states. Alpha diversity at the initial timepoint did not differ between samples that subsequently became colonized versus those that did not (Ax: $p = 0.85$; Ic: $p = 0.53$). However, beta diversity analysis revealed a modest but significant shift in community composition associated with persistence status (PERMANOVA $p = 0.036$, $R^2 = 0.0525$), without differences in dispersion ($p = 0.689$), indicating subtle compositional restructuring rather than broad diversity changes.

In contrast to *E. coli*, *S. epidermidis* showed weaker and less consistent associations with microbiome structure. Relative abundance differed modestly between colonized and non-colonized samples in a site-dependent manner, but no consistent pattern was observed between persistent and non-persistent states, with the latter group being limited in size. Alpha diversity at baseline was not associated with subsequent colonization (Ax: $p = 0.49$; Ic: $p = 0.32$). Similarly, beta diversity analysis did not reveal significant compositional differences associated with persistence status (PERMANOVA $p = 0.301$, $R^2 = 0.0586$), indicating an absence of detectable community-level restructuring in this context.

Overall, persistent carriage occurs against a broadly stable skin microbiome background. *E. coli* persistence is associated with a modest community shift dominated by E coli itself, whereas *S. epidermidis* persistence primarily reflects increased community heterogeneity rather than systematic compositional restructuring. we have added texts in the revised manuscript that includes the limitations of these analyses.

Figures below are the analyses undertaken for review purposes.

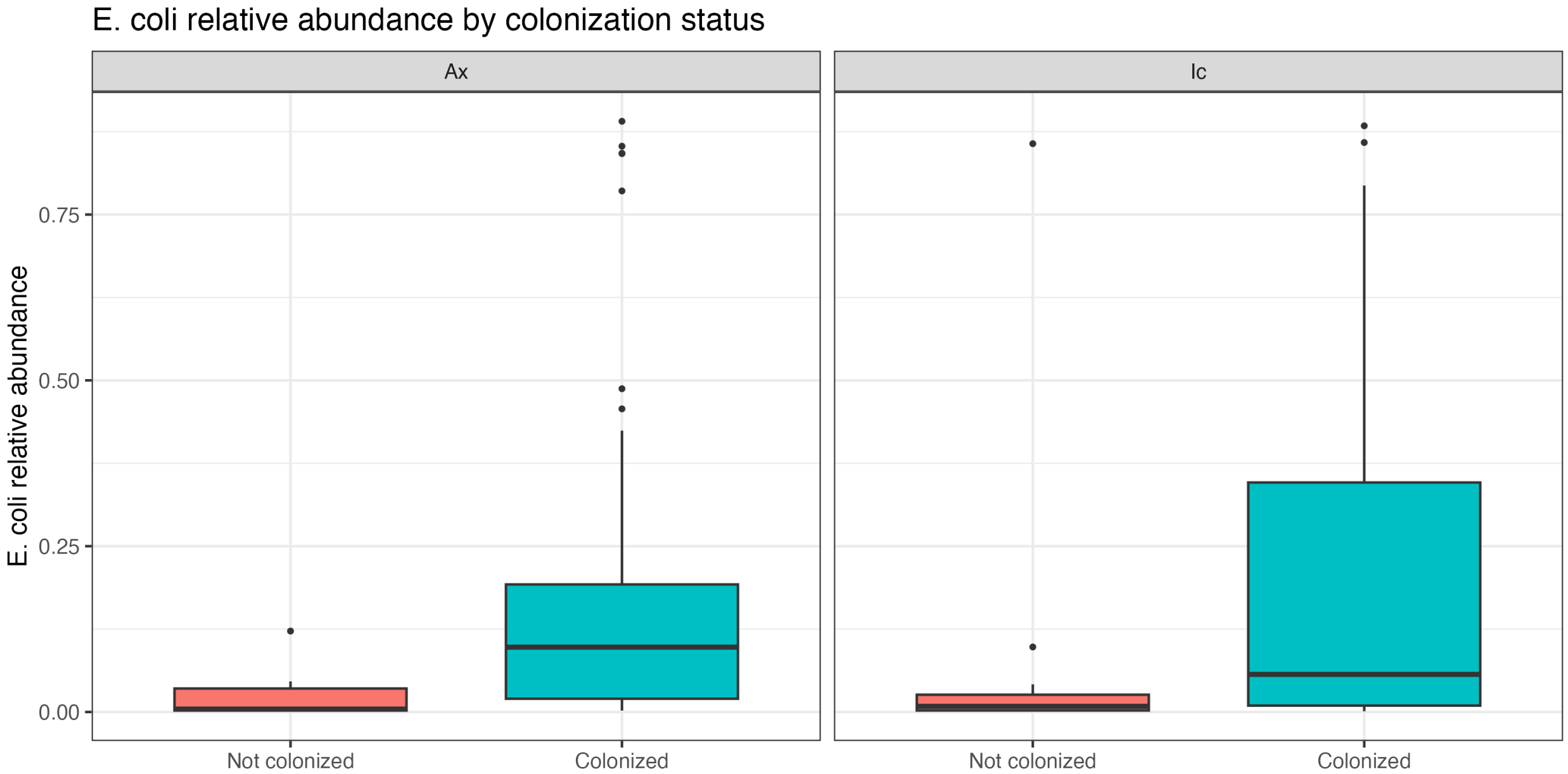
E. coli relative abundance by colonization status
Ax
Ic
E. coli relative abundance
0.00
0.25
0.50
0.75
Not colonized
Colonized
Not colonized
Colonized

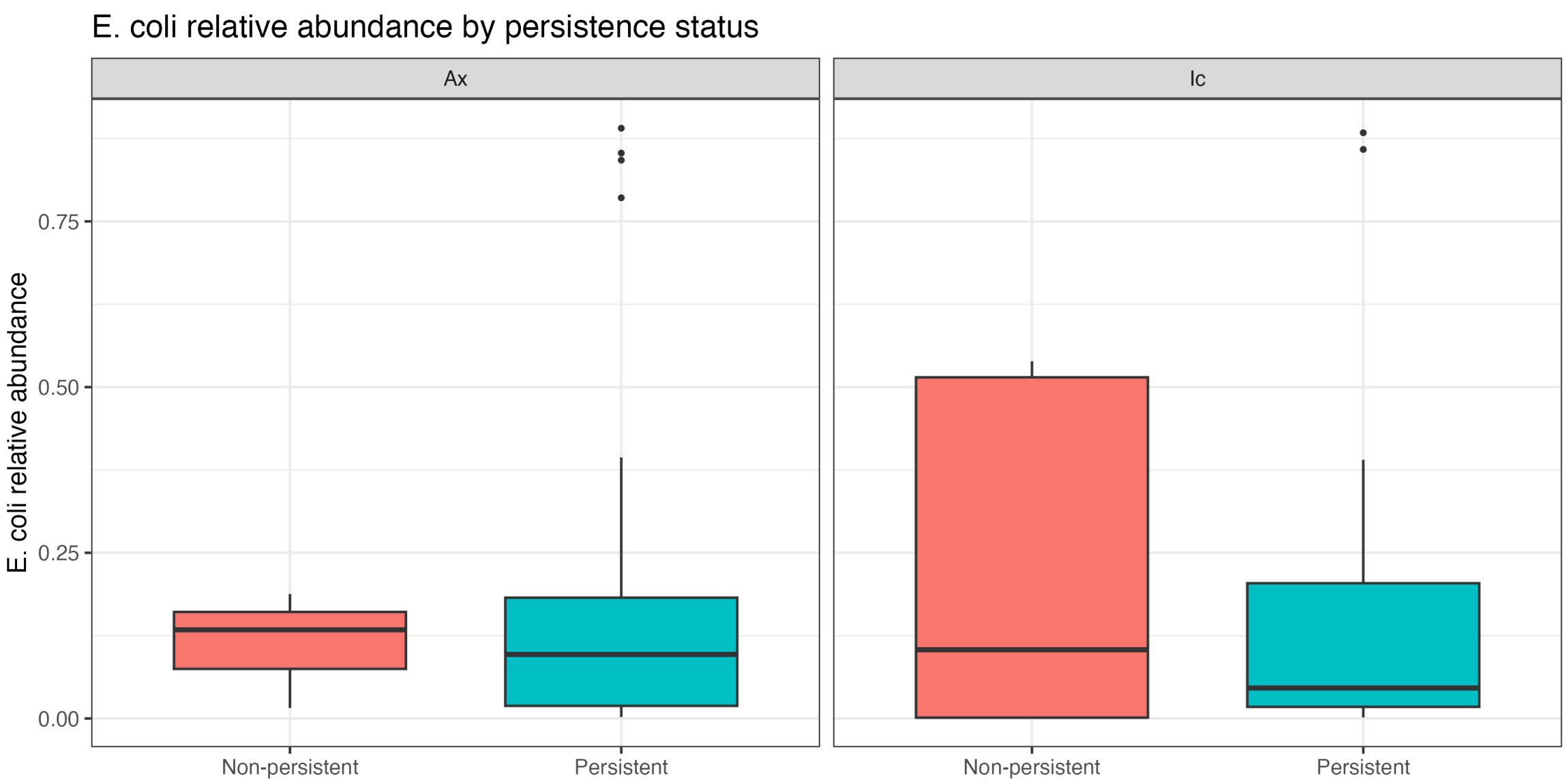
E. coli relative abundance by persistence status
Ax
Ic
E. coli relative abundance
0.00
0.25
0.50
0.75
Non-persistent
Persistent
Non-persistent
Persistent

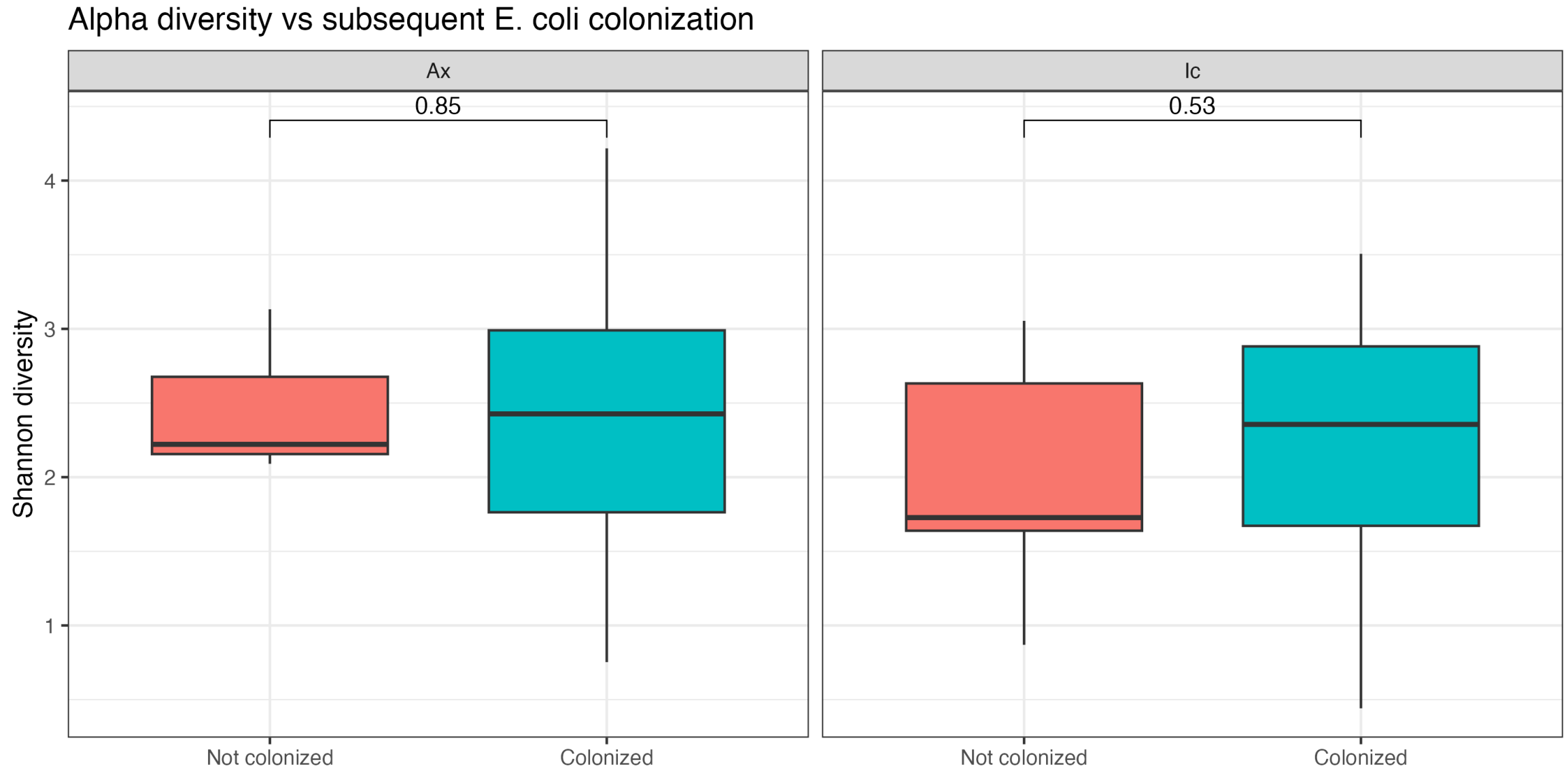
Alpha diversity vs subsequent E. coli colonization
Ax
Ic
0.85
0.53
Shannon diversity
4
3
2
1
Not colonized
Colonized
Not colonized
Colonized

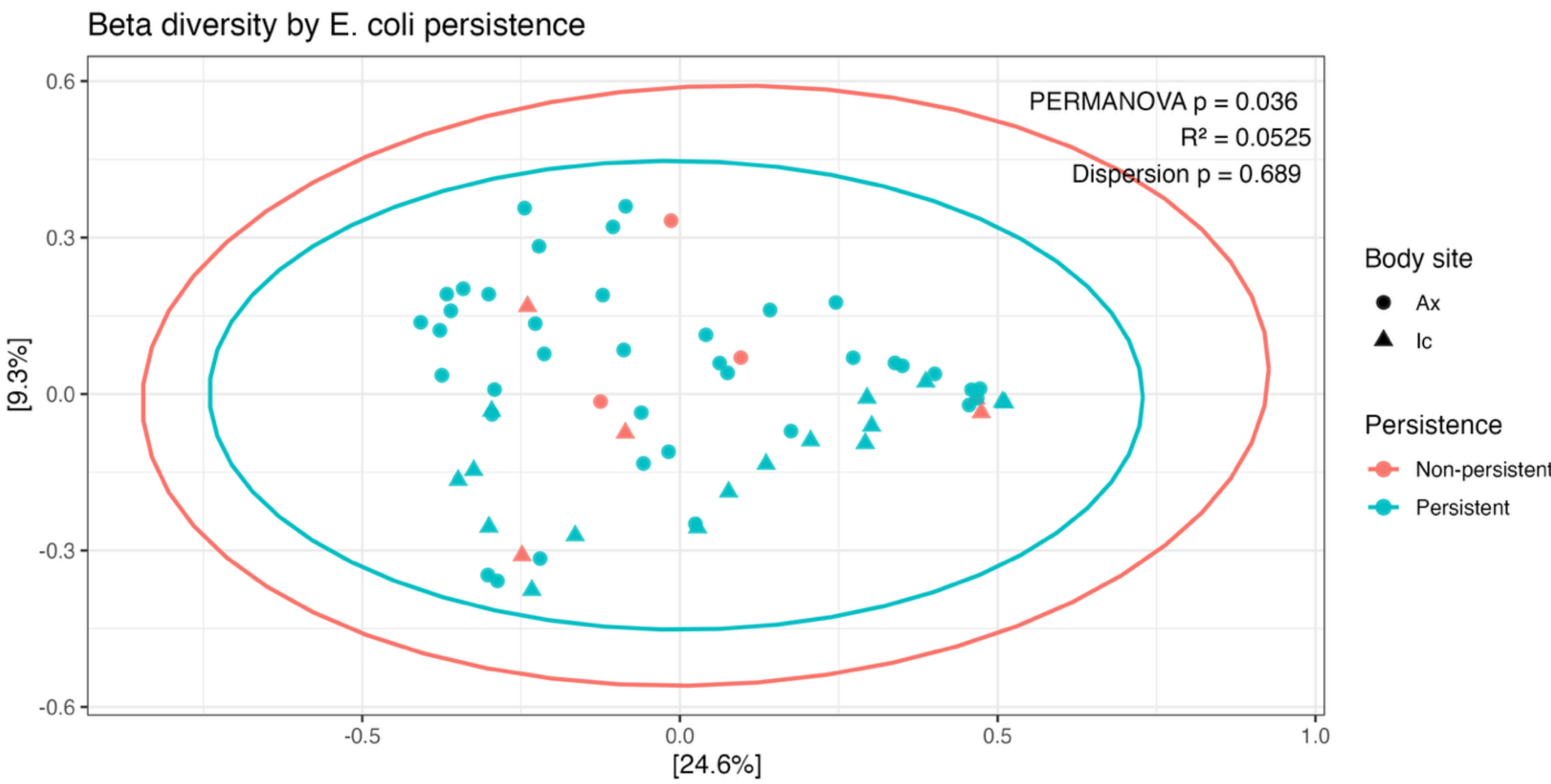
Beta diversity by E. coli persistence
PERMANOVA p = 0.036
R² = 0.0525
Dispersion p = 0.689
0.6
0.3
0.0
-0.3
-0.6
[9.3%]
-0.5
0.0
0.5
1.0
[24.6%]
Body site
Ax
Ic
Persistence
Non-persistent
Persistent

S. epidermidis relative abundance by colonization status

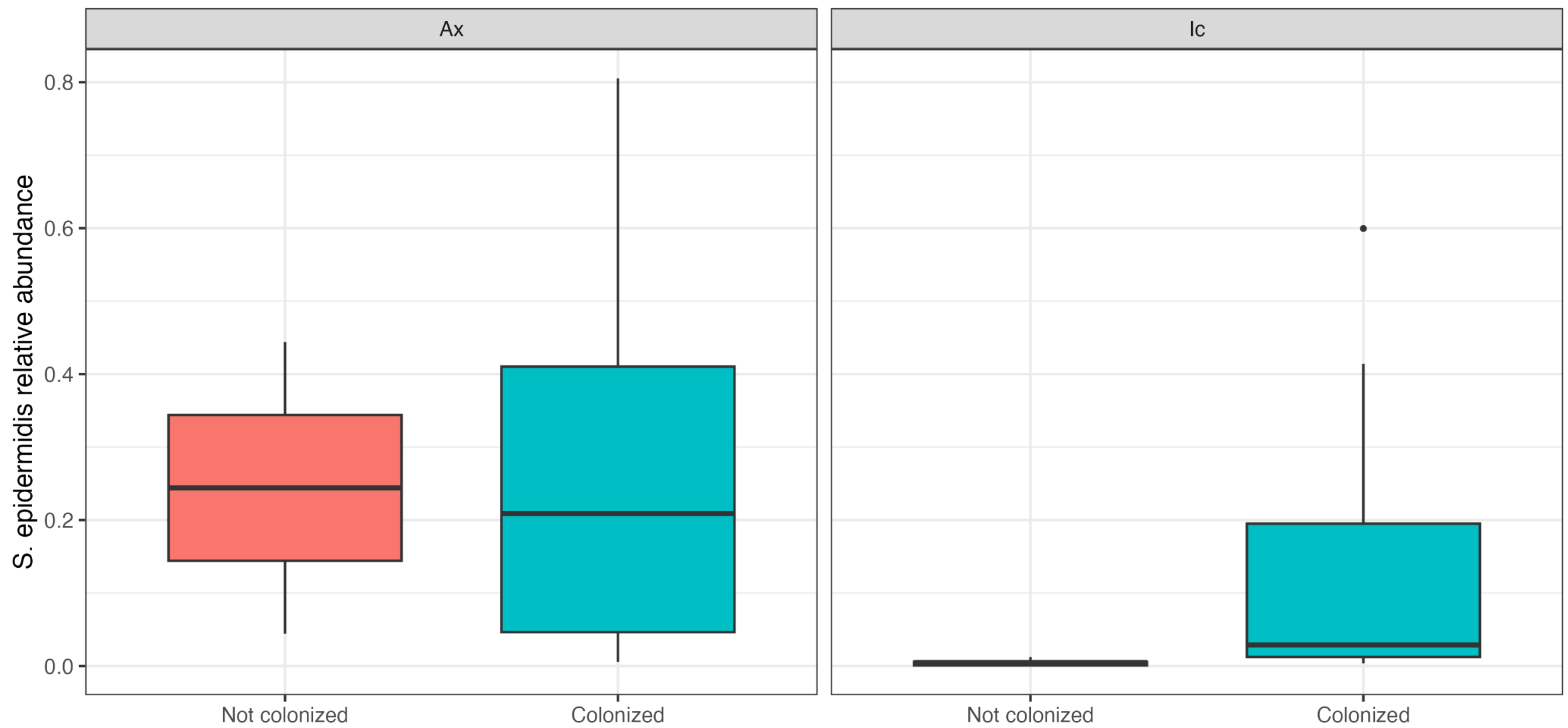

S. epidermidis relative abundance by persistence status

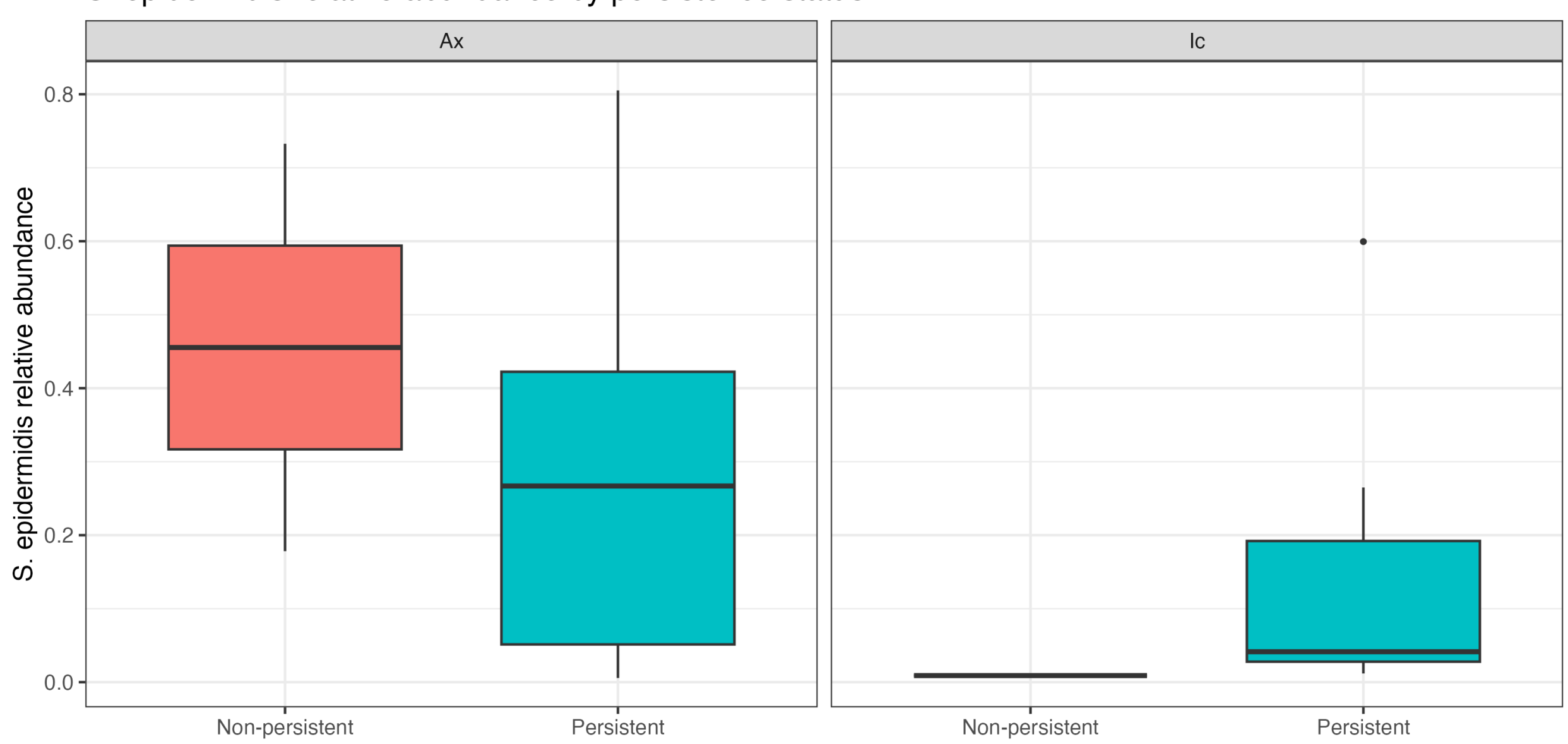

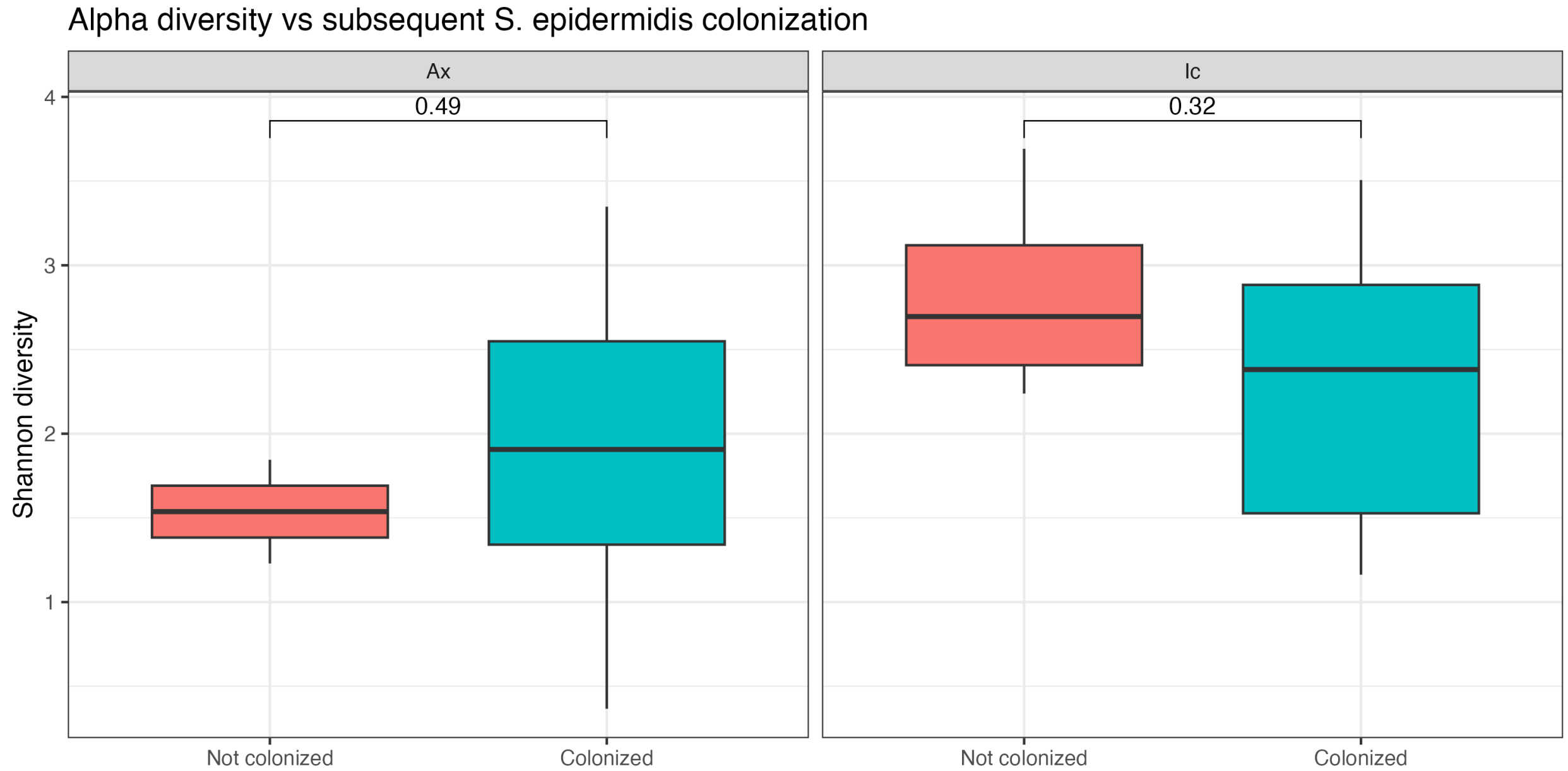

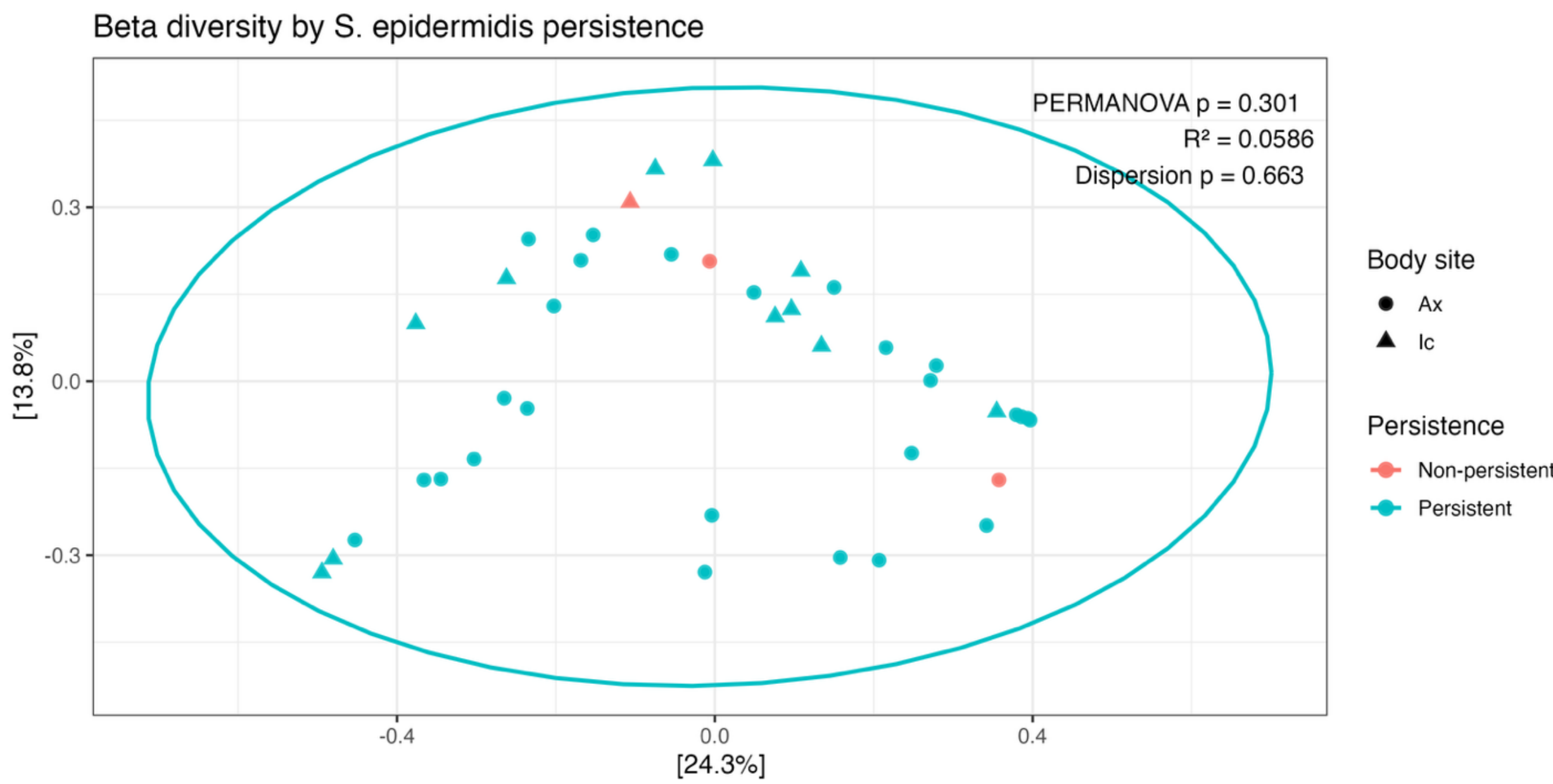

5.) The introduction of SNPs in the core genes suggests clonal expansion. It is worth to analyze if the clonally expanding E. coli 131 and S. epidermidis ST2 genome had harbored any additional accessory genes (related to anti-microbial resistance) over the spread among different residents and facility. This will add more value to this study.

We agree that assessing accessory genome dynamics in clonally expanding lineages adds more values. In our dataset, the strongest signal of regional clonal expansion was observed for *E. coli* ST93 and *S. epidermidis* ST2.

To address the reviewer's comment and determine whether the clonal expansion of *E. coli* ST93 was associated with accessory genome remodeling, we annotated genomes with Prokka and ran them on Panaroo to analyze the gene presence-absence matrix within the *E. coli* ST93 lineage. Variable genes were predominantly annotated in the accessory genome as hypothetical proteins or general metabolic and transport

functions, with occasional mobile element-related genes occurring sporadically rather than in a clone-associated pattern.

We next performed the same analysis for the clonally expanding *S. epidermidis* ST2 lineage. Genomes were annotated with Prokka and the panaroo gene presence-absence matrix was searched. Again, variable genes were predominantly annotated as hypothetical proteins or general metabolic and transport functions, with occasional mobile element–associated genes occurring sporadically rather than in a lineage-associated pattern. Importantly, we did not observe consistent acquisition of antimicrobial resistance determinants during spread. We confirm that dissemination of *S. epidermidis* ST2 in this cohort is primarily explained by clonal spread rather than ongoing accessory genome remodeling.

6.) Also, do authors have antibiotic susceptibility profile of all these clonally expanded lineages of E. coli 131 and S. epidermidis ST2 among different resistance and different nursing home settings?

The cultured *E. coli* ST131 isolates were recovered on ESBL-selective media and were therefore confirmed to exhibit an ESBL phenotype; however, comprehensive AST panels were not performed for these isolates. For the majority of detected lineages including *S. epidermidis* ST2 and metagenomically recovered *E. coli* ST93 organism recovery was culture-independent via metagenome-assembled genomes (MAGs). In these cases, antimicrobial resistance potential was inferred from genomic resistance determinants.

Reviewer #2 (Remarks to the Author):

Hounmanou et al combine microbial culturing, shotgun metagenomics and genome/metagenome assembly to show that multiple microbial strains and anti-microbial resistance genes are shared within and between nursing homes, with residents' skin serving as a reservoir for acquisition and persistence. The study further illustrates the added value of metagenomic sequencing for detecting microbes of potential clinical concern in a vulnerable population that would be difficult to capture comprehensively by culture-based approaches alone. Overall, the data support the main observations and merit publication, although several aspects of interpretation and presentation would benefit from clarification, as outlined below:

We thank the reviewer for their careful evaluation of our work and for the constructive comments provided to improve the manuscript. We are pleased that the reviewer finds the overall observations supported by the data. Below, we address each point in detail.

1. The authors posit "a two-tiered colonization landscape" of two E. coli strains: ST131(ESBL +; less prevalent) and ST93 (ESBL -; more prevalent). In rarer cases where the two strains co-exist e.g. on resident 0802, are the strain relative abundances estimated by StrainGE comparable, or does ST93 dominate the sample? By extension, are most axilla and inguinal crease samples dominated by a single lineage of E. coli? Do relative strain abundances of E. coli and species like S. epidermidis with high intra-species diversity change following bathing? These patterns would be informative for assessing whether intra-species competition limits co-colonization, as observed for other skin microbes (PMID: 40588591).

We thank the reviewer for this important suggestion. To evaluate the proposed "two-tiered colonization landscape" and assess strain-level co-colonization dynamics, we performed strain-resolved analyses using *E. coli* and *S. epidermidis* relative abundance estimates derived from StrainGE.

For *E. coli*, we observed a clear pattern of single-lineage dominance. Across Ax/Ic samples with confident detection, 97.4% (76/78) were dominated by a single lineage, with the most abundant strain accounting for nearly all of the signal (median share = 1.00). ST93 was the predominant lineage when present, while co-detection of ST93 and ST131 within the same site was rare and limited to a few low-abundance cases. In the reviewer-highlighted resident (0802), strain distributions reflected site-specific partitioning (ST93 in axilla, ST131 in inguinal crease) rather than within-site co-dominance. Importantly, dominant lineage patterns were stable across visits, including post-bathing intervals, with no systematic shifts in relative abundance. Together, these results indicate that *E. coli* colonization on skin is characterized by strong intra-species competitive structuring and near-universal single-lineage dominance.

S. epidermidis exhibited higher within-site lineage diversity. Across Ax/Ic samples, only 51.6% (47/91) were single-lineage dominant, while nearly half showed multi-lineage structure. Multiple strains were detectable within the same site in the majority of samples, including in individuals with persistent ST2 colonization. In these cases, ST2 was typically maintained over time but co-occurred with additional strains at lower relative abundance. As with *E. coli*, dominant strains were generally stable across visits, and we did not observe systematic changes in relative abundance following bathing.

Overall, these analyses support a species-specific ecological contrast: *E. coli* displays strong competitive exclusion with single-lineage dominance, whereas *S. epidermidis*, a common skin commensal, frequently exhibits multi-lineage coexistence within the same niche.

Below we provide self-descriptive figures for review purposes to support the analyses done.

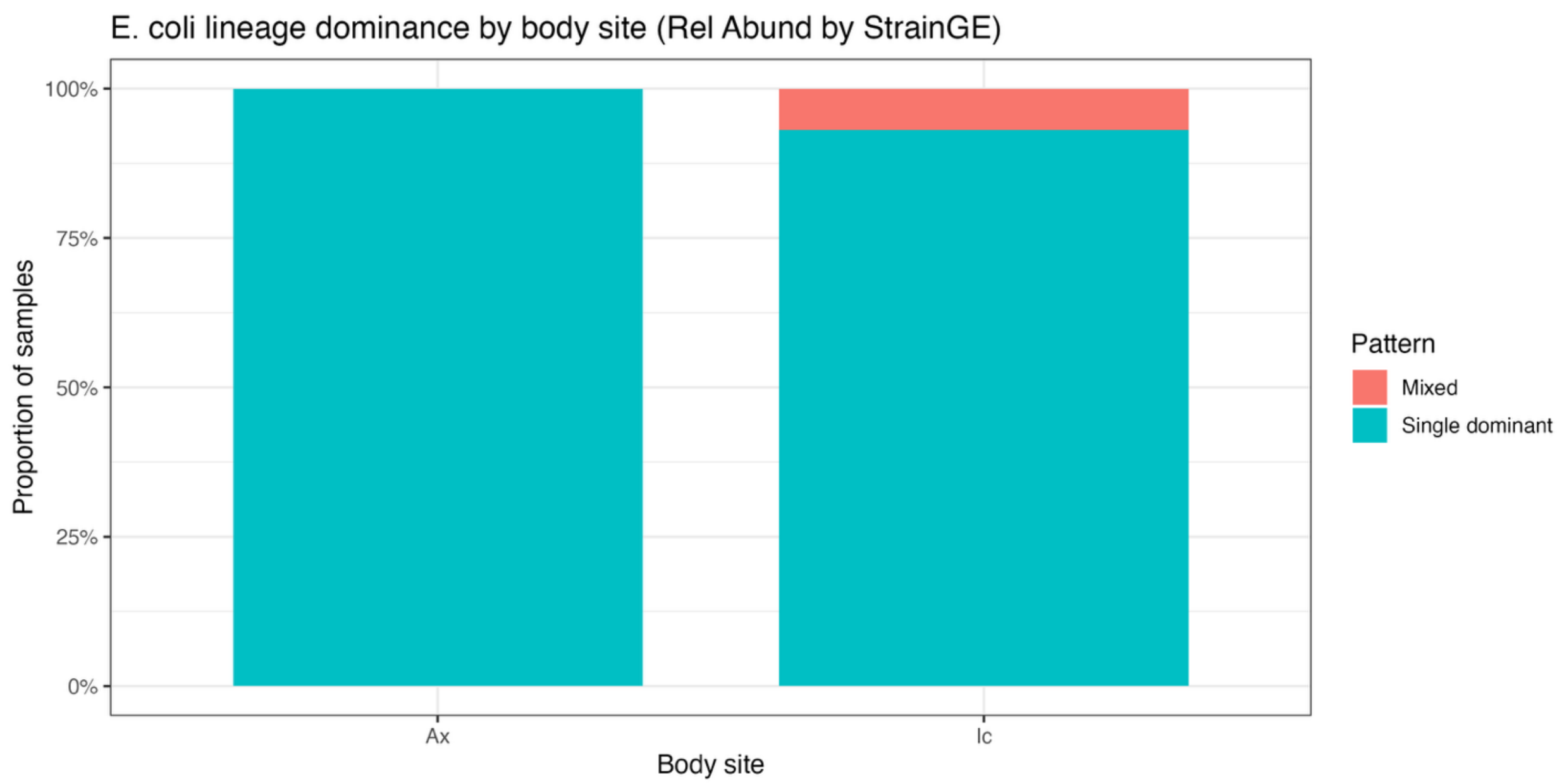
E. coli lineage dominance by body site (Rel Abund by StrainGE)
100%
75%
50%
25%
0%
Proportion of samples
Ax
Ic
Body site
Pattern
Mixed
Single dominant

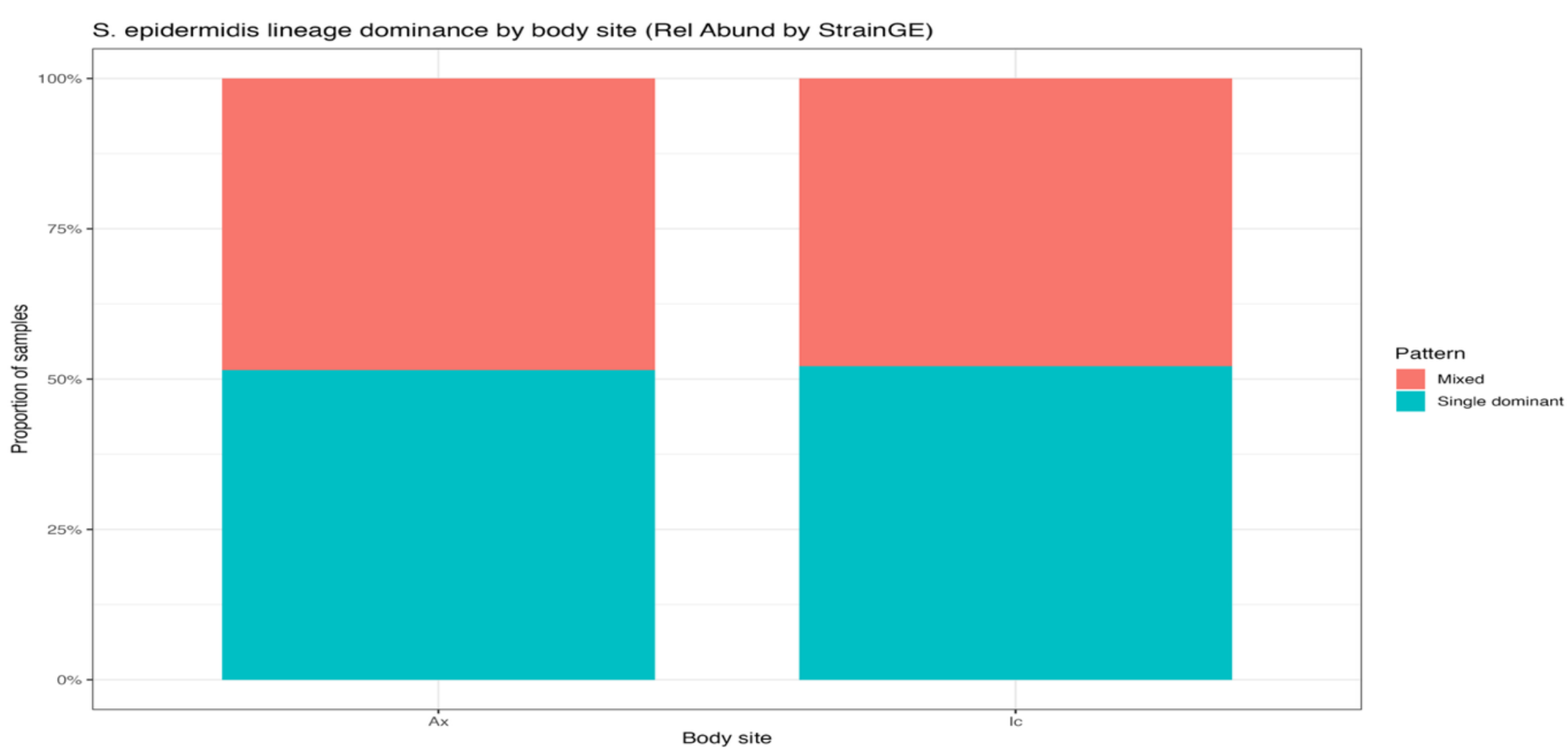
S. epidermidis lineage dominance by body site (Rel Abund by StrainGE)
100%
75%
50%
25%
0%
Proportion of samples
Ax
Ic
Body site
Pattern
Mixed
Single dominant

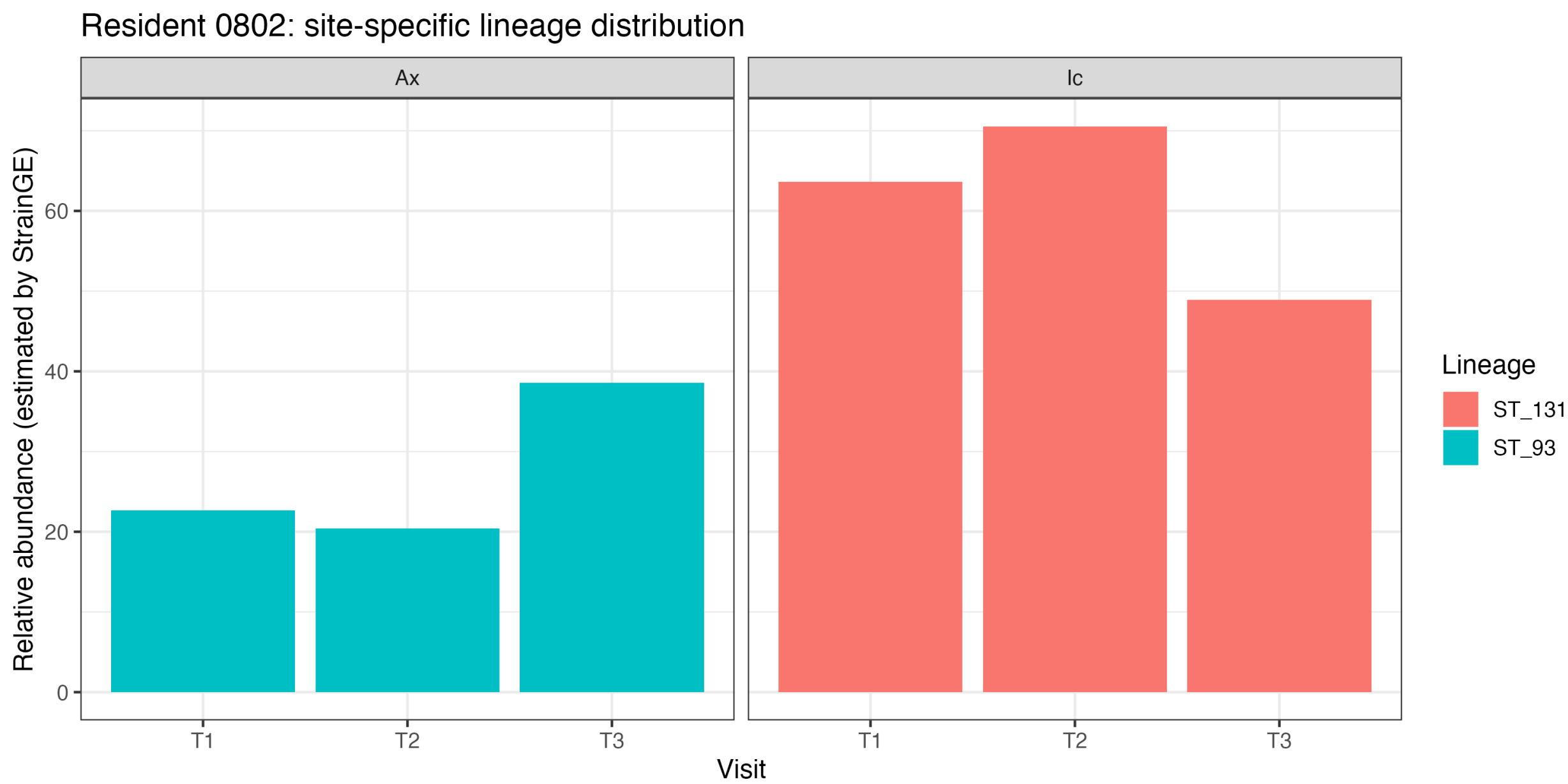
Resident 0802: site-specific lineage distribution
Ax
Ic
Relative abundance (estimated by StrainGE)
0
20
40
60
T1
T2
T3
Visit
Lineage
ST_131
ST_93

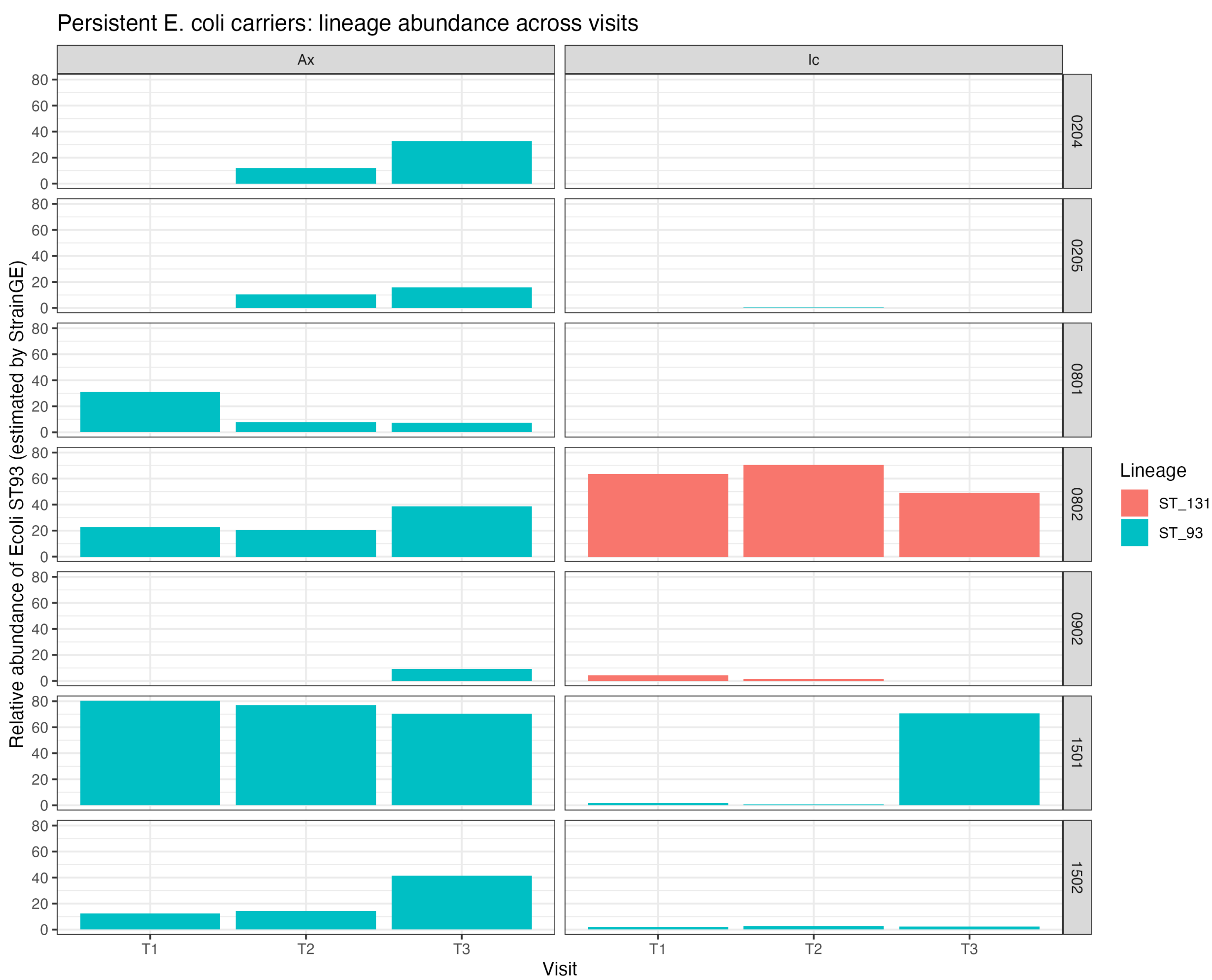
Persistent E. coli carriers: lineage abundance across visits
Ax
Ic
0204
0205
0801
0802
0902
1501
1502
Relative abundance of Ecoli ST93 (estimated by StrainGE)
0
20
40
60
80
T1
T2
T3
Visit
Lineage
ST_131
ST_93

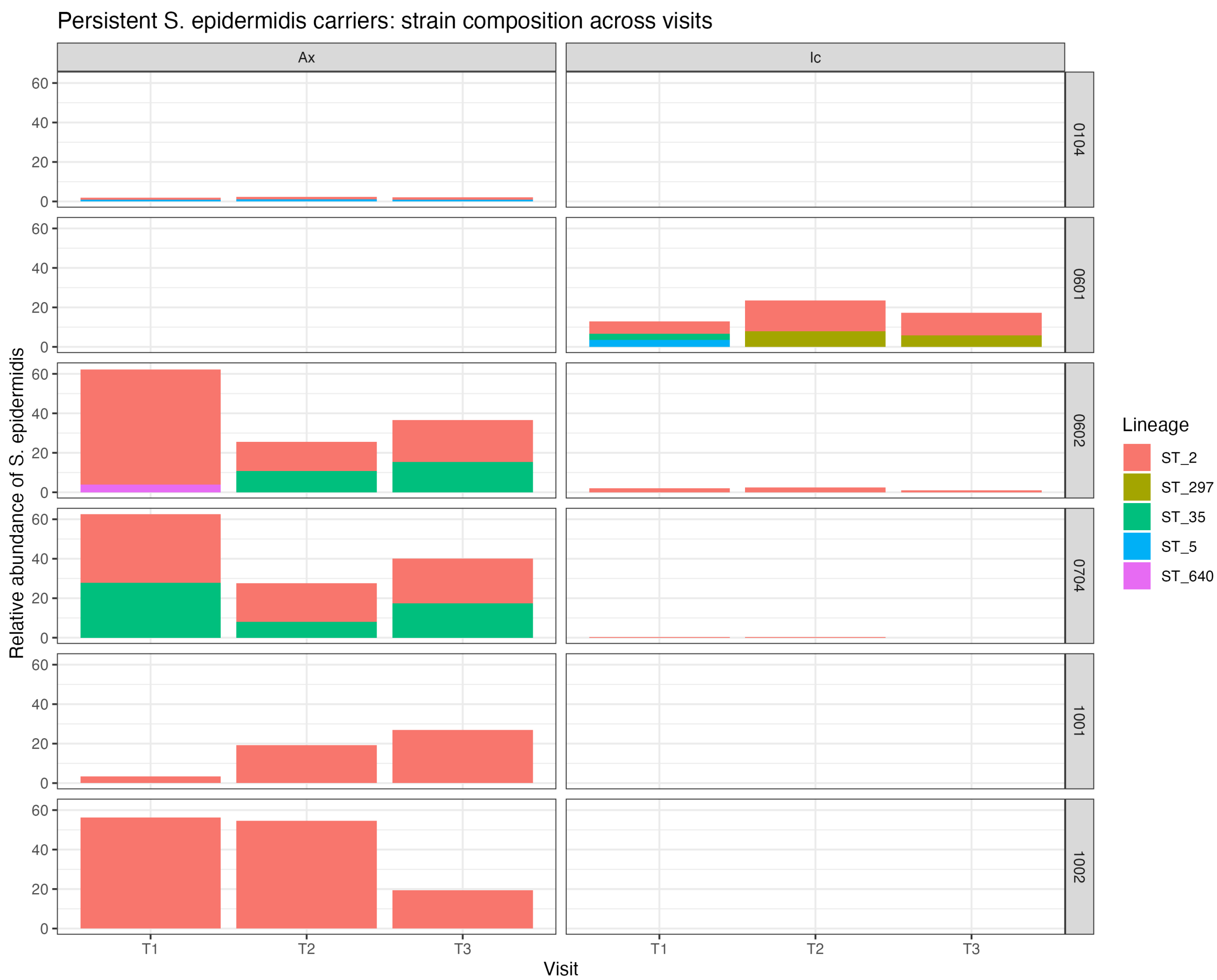
Persistent S. epidermidis carriers: strain composition across visits
Ax
Ic
0104
0601
0602
0704
1001
1002
Relative abundance of S. epidermidis
Visit
T1
T2
T3
Lineage
ST_2
ST_297
ST_35
ST_5
ST_640

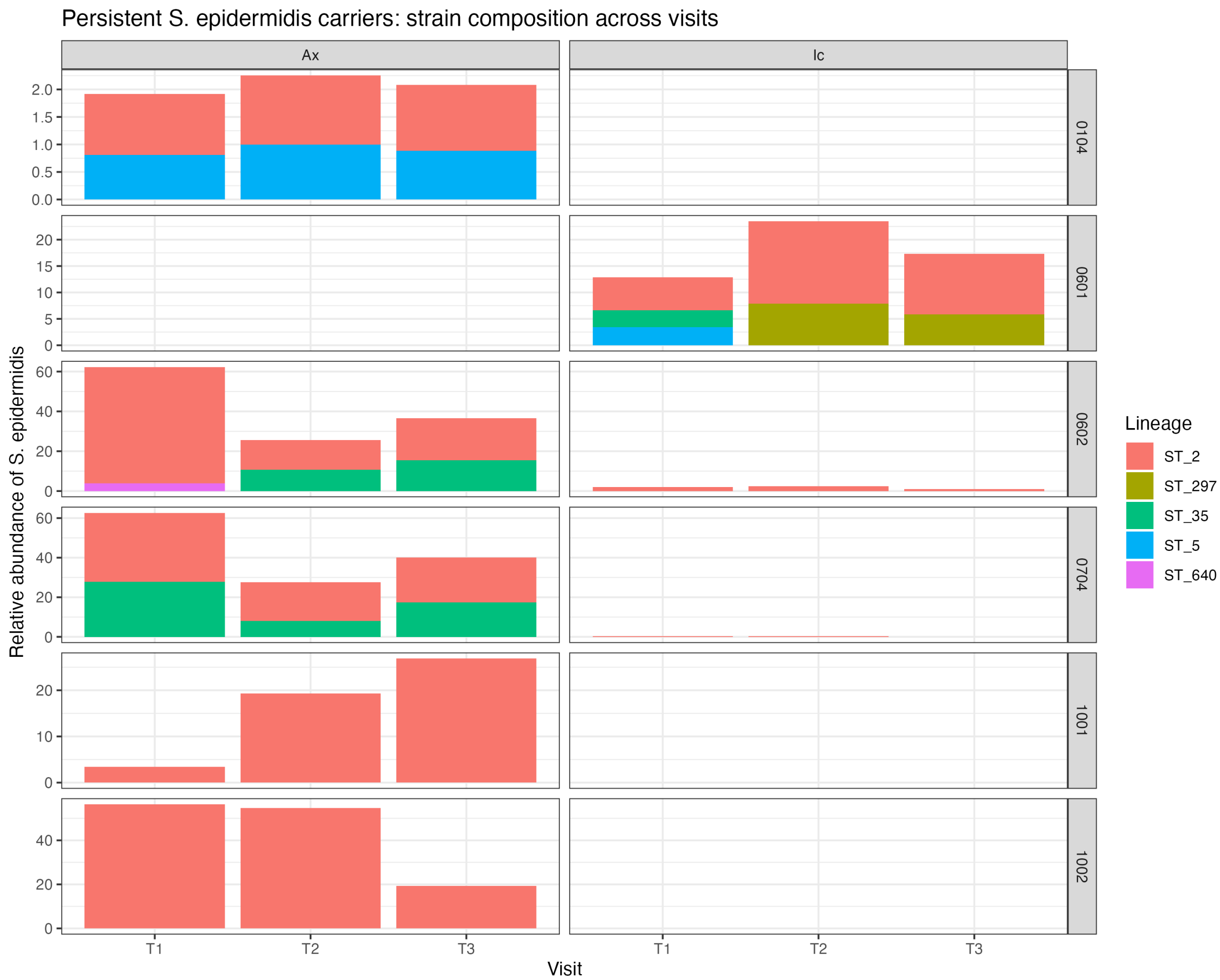

2. In several places (e.g., lines 174–176 and 191–194; Fig. 3 legends), the manuscript describes specific AMR genes as being carried by E. coli MAGs of specific strains. However, the methods indicate that these resistance determinants were detected by read-level mapping. Because many of these AMR genes are typically plasmid-borne and can circulate among multiple Proteobacteria, it is unclear how strainGE or BLASTn could confidently assign plasmid-derived contigs harboring these genes specifically to E. coli strains. This is especially relevant given that other Proteobacteria are present at these sites (Fig. 1B) and could plausibly serve as reservoirs for the detected AMR genes.

We thank the reviewer for this important point regarding assignment of AMR determinants in metagenomic data. ARGs were assessed using both metagenome-assembled genomes (MAGs) and read-level mapping. Resistance determinants were first identified from MAG annotations when present, and read-based detections using RGI against CARD were used to complement this analysis, particularly in cases where ARGs were not recovered within near-complete MAGs.

To minimize potential misattribution of plasmid-borne genes, downstream analyses were restricted to samples containing high-quality, strain-resolved MAGs and were conservatively filtered to exclude cases with co-detection of plausible alternative host species. Specifically, blaCTX-M signals were interpreted only in samples containing E. coli MAGs and lacking co-detection of other Enterobacterales (e.g., *Proteus mirabilis*). Similarly, mecA was attributed to *S. epidermidis* only in samples with corresponding high-quality *S. epidermidis* MAGs and without evidence of *S. aureus*. As an additional safeguard, assembled contigs

harboring key resistance determinants were inspected by BLASTn and evaluated alongside StrainGE profiling, which consistently supported *E. coli* as the dominant Proteobacterial host in the analyzed samples. These ARG-carrying contigs were generally short to intermediate in length and did not consistently include sufficient flanking sequence to confidently distinguish chromosomal versus plasmid origin, although in some cases neighboring genes were consistent with Enterobacterales genomic context.

While we cannot fully exclude plasmid mobility inherent to metagenomic inference, particularly for ARG classes such as blaCTX-M that are frequently plasmid-associated**,** these conservative, multi-layered filters substantially reduce the likelihood of cross-species misassignment. We did not perform dedicated plasmid reconstruction (e.g., via MOB-suite), as our objective was conservative host attribution rather than resolution of mobile genetic element architecture. We have clarified this workflow and its limitations in the revised Methods.

3. The authors make multiple references to "strain transmission dynamics" (e.g. lines 82 and 297) in the manuscript. However, aside from within-individual bathing comparisons, the analyses of inter-facility spread are cross-sectional and do not track the movement of specific strains between individuals or facilities over time. As such, it would be helpful to clarify whether the authors are referring to ' transmission dynamics' in a temporal sense, or to adjust the terminology to better reflect the static nature of the available data for cross-site analysis.

We thank the reviewer for this clarification. We agree that our dataset does not directly track person-to-person transmission events over time across facilities. In this study, the term "transmission dynamics" was intended to describe patterns of strain sharing and genomic relatedness consistent with potential dissemination, rather than to infer temporally resolved transmission pathways between individuals or nursing homes.

Our longitudinal sampling supports short-term within-host persistence following bathing, whereas inter-resident and inter-facility comparisons are based on high-resolution genomic similarity at cross-sectional timepoints. To avoid overinterpretation, we have revised the text throughout the manuscript to more precisely describe these findings as evidence of strain sharing and regional dissemination patterns rather than directly observed transmission events. We have also clarified this distinction in the Results and Discussion.

4. Line 436: The authors write that "Culturing confirmed that these MDROs were viable and replicating on the skin…" This over-interprets the data, as most species and strains detected by metagenomics were not successfully cultured in this study. While microbial recovery post-bathing provides indirect support for viability, it could also reflect re-seeding from the environment. To strengthen claims about active replication, the authors could consider computational approaches such as iRep (PMCID: PMC5538567) or GriD (PMID: 30470746) to estimate growth rates of specific species from metagenomic data.

We thank the reviewer for this helpful suggestion and agree that our original wording overstated the evidence for active replication.

Culture-based isolation in this study confirms viability for a subset of MDROs detected on resident skin (including ESBL-producing *E. coli* ST131, Proteus mirabilis and Providencia, VRE, and MRSA), although not all metagenomically detected organisms were cultured.

To further evaluate replication, we implemented the computational growth-rate estimator iRep analysis. Across dominant lineages including *E. coli* ST93, *E. coli* ST131, and *S. epidermidis* ST2 iRep values were consistently ≥1, supporting ongoing replication rather than strict dormancy (see iREP output Tables provided for review purposes). We recognize the limitations of coverage-based growth inference in low-biomass skin samples; therefore, we interpret these results as supportive evidence of biological activity rather than definitive proof of rapid proliferation.

5. Line 283: "Asterix" should be asterisk.
This is corrected

6. Panels in figure 7 should be more clearly labelled as to which species are being analysed, because C-D, E-F and G-H relate to three different species.

This has been corrected in the legends

7. Line 536: "Full computational workflows, detailed software parameters, command-line scripts and codes are provided on github.com/skinmicrobiome." For clarity, please change the link to specific github repository for this project.

The scripts now been added to the correct repository within the lab's github with the link below:
https://github.com/skinmicrobiome/2026_Hounmanou_NursingHome

Reviewer #3 (Remarks to the Author):

I co-reviewed this manuscript with one of the reviewers who provided the listed reports. This is part of the Nature Communications initiative to facilitate training in peer review and to provide appropriate recognition for Early Career Researchers who co-review manuscripts.

We thank the reviewers for this coordinated effort.

REVIEWERS' COMMENTS

Reviewer #1 (Remarks to the Author):

The authors addressed the comments. no further concerns.

Response

We thank the reviewers for the contribution

Reviewer #2 (Remarks to the Author):

The authors have fully addressed my concerns raised in the initial review, with appropriate revisions and additional analyses in the rebuttal document. I support the publication of the revised work.

Response

We thank the reviewers for the contribution

Reviewer #3 (Remarks to the Author):

I co-reviewed this manuscript with one of the reviewers who provided the listed reports. This is part of the Nature Communications initiative to facilitate training in peer review and to provide appropriate recognition for Early Career Researchers who co-review manuscripts.

Response

We thank the reviewers for the contribution
